# Flexible and high-performance electrochromic devices enabled by self-assembled 2D TiO_2_/MXene heterostructures

**DOI:** 10.1038/s41467-021-21852-7

**Published:** 2021-03-11

**Authors:** Ran Li, Xiaoyuan Ma, Jianmin Li, Jun Cao, Hongze Gao, Tianshu Li, Xiaoyu Zhang, Lichao Wang, Qinghong Zhang, Gang Wang, Chengyi Hou, Yaogang Li, Tomás Palacios, Yuxuan Lin, Hongzhi Wang, Xi Ling

**Affiliations:** 1 State Key Laboratory for Modification of Chemical Fibers and Polymer Materials, College of Materials Science and Engineering, Donghua University, Shanghai, China; 2 Department of Chemistry, Boston University, Boston, MA USA; 3 Division of Materials Science and Engineering, Boston University, Boston, MA USA; 4 Department of Electrical Engineering and Computer Science, Massachusetts Institute of Technology, Cambridge, MA USA; 5 The Photonics Center, Boston University, Boston, MA USA; 6 Present Address: Department of Electrical Engineering and Computer Sciences, University of California, Berkeley, CA USA

**Keywords:** Two-dimensional materials, Electronic devices

## Abstract

Transition metal oxides (TMOs) are promising electrochromic (EC) materials for applications such as smart windows and displays, yet the challenge still exists to achieve good flexibility, high coloration efficiency and fast response simultaneously. MXenes (e.g. Ti_3_C_2_T_x_) and their derived TMOs (e.g. 2D TiO_2_) are good candidates for high-performance and flexible EC devices because of their 2D nature and the possibility of assembling them into loosely networked structures. Here we demonstrate flexible, fast, and high-coloration-efficiency EC devices based on self-assembled 2D TiO_2_/Ti_3_C_2_T_x_ heterostructures, with the Ti_3_C_2_T_x_ layer as the transparent electrode, and the 2D TiO_2_ layer as the EC layer. Benefiting from the well-balanced porosity and connectivity of these assembled nanometer-thick heterostructures, they present fast and efficient ion and electron transport, as well as superior mechanical and electrochemical stability. We further demonstrate large-area flexible devices which could potentially be integrated onto curved and flexible surfaces for future ubiquitous electronics.

## Abstract (web summary)

Though two-dimensional (2D) nanocomposite materials have been attractive for flexible electrochromic (EC) devices, achieving both high performance and durability remains a challenge. Here, the authors report high-performance, environmentally stable 2D MXene/transition metal oxide-based EC devices.

## Introduction

Electrochromic (EC) materials, which can change their optical absorption reversibly due to the electrochemical redoxation with inserted ions under an applied potential^1–3^, have shown great promise in applications such as displays, smart windows and optical camouflage^3–5^. With the growing interest in bringing electrochromism to the field of flexible and wearable electronics in recent years, incorporating the functionality of flexibility to EC devices while maintaining high energy efficiency, fast switching speed and long durability has become increasingly critical^6,7^. However, this is an extremely challenging task because: (a) both the EC performance and the mechanical properties are nontrivially correlated with the materials of choice, their nanoscale formations, and the assembly approach; and (b) the co-optimization of the transparent conductive electrode and the EC material, as well as the way to integrate them, are needed.

Recently, two-dimensional (2D) transition metal carbides, nitrides, and carbonitrides (referred to as MXenes) have emerged as promising nanoscale electronic materials with good electrochemical stability^8,9^. They have also been reported as promising templates for the synthesis of functional micro/nanostructured materials, such as transition metal oxides (TMOs), alkali-metal titanates, and their hybrids^10,11^. Owing to their unique optical and electrical properties, MXenes and their derivatives have demonstrated their potential in the application of energy storage, catalysis, and electronics^12–14^. More importantly, 2D nanosheets of both MXenes and their derivatives can be produced in liquid phase scalably, which makes it easy for constructing self-assembled films and hybrid structures, thus suitable for device applications in large scale.

We propose that self-assembled MXene/TMO heterostructures, with the MXenes as the flexible transparent electrode, and the TMOs (derived from the MXenes) as the flexible EC layer, are promising for high performance and flexible EC devices, yet it has never been explored previously. In addition to the advantage of the easiness of fabrication and integration with different functional components from MXene derivatives as mentioned above, an even more important reason is that the nanosheets network of TMOs, such as tungsten oxide (WO_3_) and titanium oxide (TiO_2_), can potentially realize not only superior EC properties, but also reliable mechanical and chemical stability among various candidates of EC materials. First, these inorganic materials are long-lasting and robust against environmental factors such as high temperature, ultraviolet radiation, and mechanical wear^15–17^, as compared to their organic counterparts^18^. Second, the nanostructured TMO network, especially in the form of 2D nanosheets, improves electrical and ionic transport significantly, because: (a) the increased surface-to-volume ratio of nanosheets as compared to the bulk forms leads to larger interface areas with the electrolyte and the shorter ion diffusivity pathways within the nanostructured channels;^3,15^ and (b) the 2D formation of the nanostructures makes the contact area between adjacent nanosheets much larger than that between 1D or 0D structures (nanowires or quantum dots)^19^, which promotes the internanosheets electron transfer^20^. Finally, networks of 2D nanostructures also help to redistribute the induced strain evenly, thus improving both the mechanical strength and the flexibility of the film, which is fundamentally more advantageous than 1D or 0D nanostructures for flexible devices^21^.

As a proof-of-concept demonstration, we successfully assemble Ti_3_C_2_T_x_ (T = O, OH, or F) and the derived TiO_2_ nanosheets into uniform nanometer-thick films at a liquid-liquid interface, and fabricate EC devices based on the TiO_2_/Ti_3_C_2_T_x_ heterostructures on a flexible substrate. Due to the much improved ion diffusion and electron conductivity of assembled single-flake-layer TiO_2_ film and the 2D nature of both the MXene electrode and the TiO_2_ EC layer, our devices are among the best flexible EC devices with the fastest coloration speed, the highest coloration efficiency and superior electrochemical stability. We further demonstrate a large-area flexible device which could potentially be integrated with arbitrary curved and flexible surfaces for future ubiquitous electronics.

## Results

### Preparation of 2D TiO_2_ via the chemical conversion from Ti_3_C_2_T_x_

**Fig. 1:**
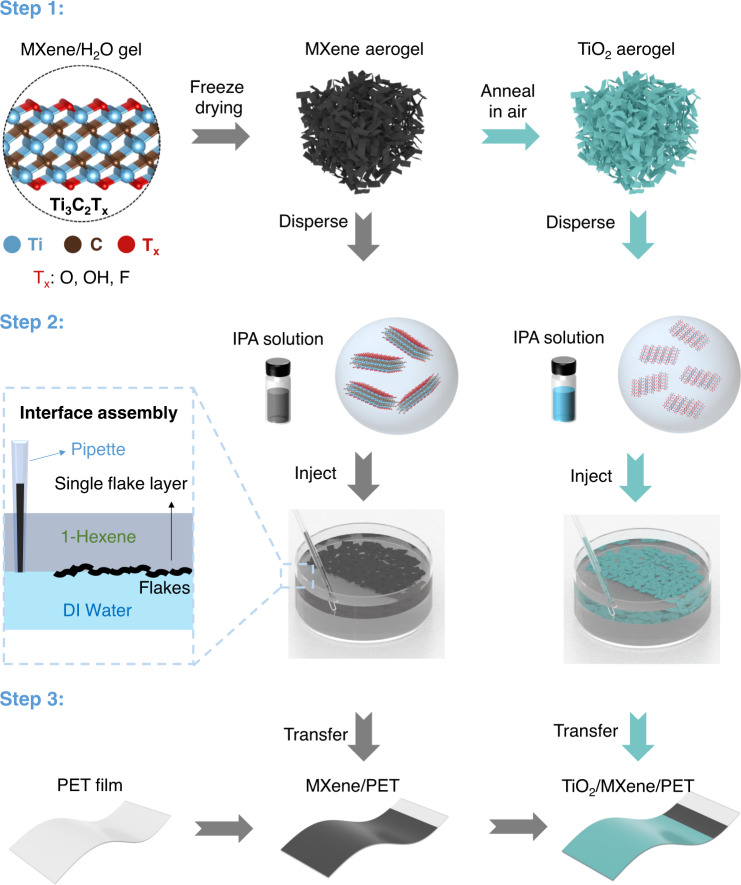
Fabrication process of TiO_2_/Ti_3_C_2_T_x_ heterostructure on a flexible substrate. Step 1: MXene (Ti_3_C_2_T_x_, crystal structures in the left panel, is drawn by Vesta software) aerogel is obtained by freeze drying of the Ti_3_C_2_T_x_ nanosheets dispersion and the derived TiO_2_ aerogel is obtained by annealing the MXene aerogel in air at 400 °C. Step 2: Ti_3_C_2_T_x_ and TiO_2_ 2D nanosheets are dispersed, respectively, in IPA, injected into the interface between hexene and DI water, and self-assembled into nanometer-thick Ti_3_C_2_T_x_ and TiO_2_ films. Step 3: the thin films are transferred onto a flexible substrate (e.g., PET) in a layer-by-layer fashion to realize the TiO_2_/MXene heterostructure, where the TiO_2_ layer serves as the electrochromic component, and the MXene layer serves as the transparent electrode.

Figure 1 illustrates the fabrication process of the self-assembled TiO_2_/Ti_3_C_2_T_x_ heterostructure on a flexible structure as the core functional component of EC devices. The fabrication process consists of three steps: First, the Ti_3_C_2_T_x_ nanosheet dispersion in deionized (DI) water is obtained from selective etching of Ti_3_AlC_2_ powder (MAX phase)^22^, and diluted to 0.2 mg/L, followed by the formation of the Ti_3_C_2_T_x_ aerogel through freeze drying. The Ti_3_C_2_T_x_ aerogel is then annealed in air at 400 °C to fully be converted to the TiO_2_ aerogel; Second, the Ti_3_C_2_T_x_ (TiO_2_) aerogels are dispersed into isopropyl alcohol (IPA) to form the Ti_3_C_2_T_x_ (TiO_2_) solutions, which are then injected into the interface between hexane and water, where the nanosheets self-assemble into uniform nanometer-thick films; Last, the self-assembled Ti_3_C_2_T_x_ film and TiO_2_ film are transferred sequentially onto a flexible polyethylene terephthalate (PET) substrate to form TiO_2_/Ti_3_C_2_T_x_ heterostructures, in which the Ti_3_C_2_T_x_ film serves as the transparent conductive electrode, and the TiO_2_ film serves as the EC layer in EC devices (more details about film assembly and transfer processes are in Supplementary Fig. 1, Supplementary Movie 1 and Supplementary Movie 2 in Supplementary Information).

**Fig. 2:**
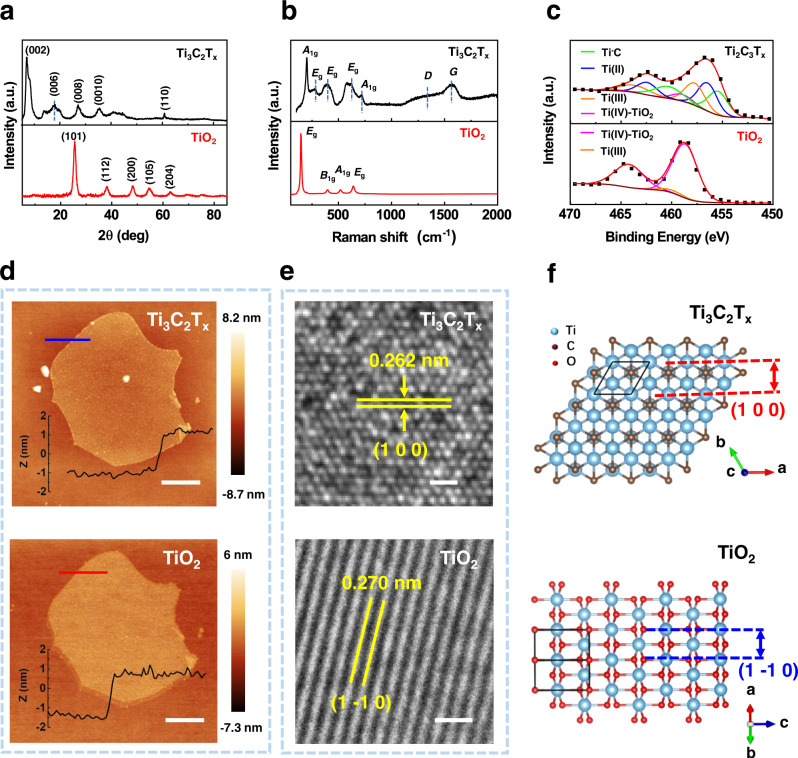
Conversion of Ti_3_C_2_T_x_ into 2D TiO_2_ flakes. **a** XRD patterns and **b** Raman spectra of the Ti_3_C_2_T_x_ before and after annealing at 400 °C for 50 h. **c** XPS spectra of the Ti_3_C_2_T_x_ and the derived TiO_2_; **d** AFM images and height profiles of Ti_3_C_2_T_x_ and a derived TiO_2_ flake on a SiO_2_/Si substrate (scale bar: 2 μm); **e** HRTEM images of Ti_3_C_2_T_x_ and derived TiO_2_ flakes (scale bar: 1 nm); **f** Crystal structures of Ti_3_C_2_T_x_ and derived anatase phase of TiO_2_.

To obtain the anatase phase of TiO_2_, which has been demonstrated a good EC material^23^, we anneal the Ti_3_C_2_T_x_ aerogel in air at 400 °C for 50 h. X-ray diffraction (XRD) and Raman spectroscopy of the flakes are measured before and after the thermal annealing process to investigate the compositional and the structural changes. As shown in Fig. 2a, after annealing in air at 400 °C, new peaks at 26°, 38°, 48°, 54°, and 63° appear in the XRD spectra, corresponding to the (101), (112), (200), (105), and (204) planes of the anatase phase of TiO_2_^24^. Raman spectra of Ti_3_C_2_T_x_ flakes after annealing confirms the conversion from Ti_3_C_2_T_x_ to TiO_2_ (Fig. 2b), where peaks at 145 cm^−1^ (*E*_g_), 398 cm^−1^ (*B*_1g_), 518 cm^−1^ (*B*_1g_), and 639 cm^−1^ (*E*_g_) match well with the Raman features of the anatase phase of TiO_2_^25^. X-ray photoelectron spectroscopy (XPS) characterization further suggests the transformation from Ti_3_C_2_T_x_ to TiO_2_. It’s clearly seen that the peaks for the Ti element in Ti_3_C_2_T_x_ (i.e., Ti-C 2*p*_3/2_ (455.3 eV), Ti(II) 2*p*_3/2_ (456.1 eV), Ti(III) 2*p*_3/2_ (457.2)) disappear after the annealing (Fig. 2c top and Supplementary Fig. 2), while peaks at 458.8 eV and 464.8 eV, corresponding to Ti(IV) 2*p*_3/2_ and Ti(IV) 2*p*_1/2_ in TiO_2_, become dominant (Fig. 2c bottom)^26^.

To evaluate the morphology and crystallinity of the obtained TiO_2_ nanosheets, optical microscopy (OM), transmission electron microscopy (TEM) and atomic force microscopy (AFM) are used to characterize Ti_3_C_2_T_x_ flakes before and after the in situ conversion to TiO_2_. Supplementary Fig. 3 shows OM images of a typical flake (~7 µm in lateral dimension) undergoing the annealing treatment at 400 °C for 50 h, where the morphology of the flake maintains during the process. AFM images (Fig. 2d) show that the derived TiO_2_ flake remains the same shape as the Ti_3_C_2_T_x_ precursor. The surface roughness of the flake also does not change much after conversion (root mean square roughness changes from 514.3 to 528.2 pm). The thickness of the flake stays about 2 nm after being converted to TiO_2_. Typical TEM images (Fig. 2e and Supplementary Fig. 4) and selective area electron diffraction (SAED) patterns (insets of Supplementary Fig. 4) indicate that both Ti_3_C_2_T_x_ and TiO_2_ are in high crystallinity. Ti_3_C_2_T_x_ shows a lattice spacing of 0.26 nm, corresponding to its (100) plane. After annealing at 400 °C, the derived TiO_2_ flake shows a lattice spacing of 0.27 nm, which refers to the^1–10^ plane of the anatase phase of TiO_2_.

Moreover, when applying the conversion to the Ti_3_C_2_T_x_ aerogels, remained morphology is also observed after annealing at 400 °C to convert it to TiO_2_ (Supplementary Fig. 5). The pore size distribution in the Ti_3_C_2_T_x_ and TiO_2_ aerogels are similar (both have peaks at around 2 nm extracted from the nitrogen adsorption isotherm at 77K) (Supplementary Fig. 6). This result further suggests that the conversion reaction is confined on initial 2D Ti_3_C_2_T_x_ flakes, leading to the good separation of each flake in the skeleton of aerogels during the conversion process.

Although the crystal lattice is transformed drastically from hexagonal (Ti_3_C_2_T_x_) to tetragonal (anatase TiO_2_), our results clearly demonstrate the successful production of Ti_3_C_2_T_x_ flakes and their derived TiO_2_ flakes, both of which are high-quality crystals over micrometer large area with nanometer thin thicknesses. Based on these experimental results and comparing the crystal structures of Ti_3_C_2_T_x_ and the anatase phase TiO_2_, we propose the following mechanism to interpret the transformation from Ti_3_C_2_T_x_ to TiO_2_. As shown in Fig. 2f, during the conversion, two O atoms need to replace one C atom sandwiched between two adjacent Ti layers. In this process, due to the gentle gas-solid reaction, the diffusion of Ti atoms is limited, and due to the similarity of the Ti frameworks in the Ti_3_C_2_T_x_ and TiO_2_ crystals, it does not require large rearrangement of Ti atoms during the conversion^27^. Thus, the morphology of the flake can be retained.

### Self-assembled ultrathin MXene films as high-performance transparent electrodes

**Fig. 3:**
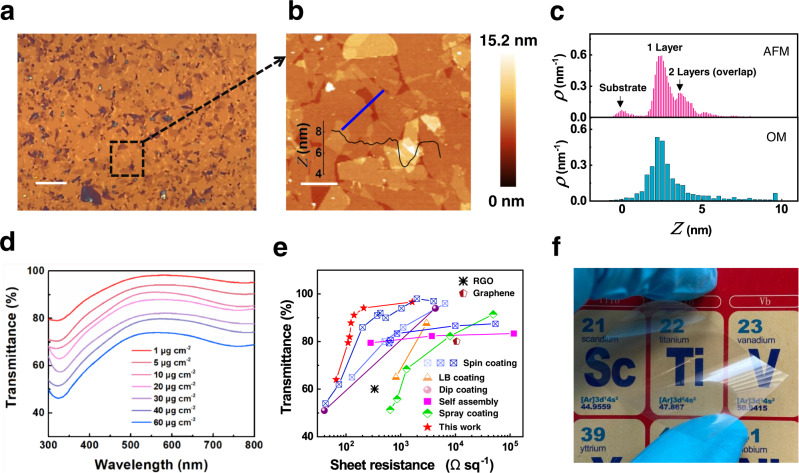
Characterization of the self-assembled Ti_3_C_2_T_x_ thin film as a transparent electrode. **a** OM image of the assembled Ti_3_C_2_T_x_ thin film (scale bar: 10 μm); **b** AFM image and the height profile of the assembled Ti_3_C_2_T_x_ thin film in corresponding area marked in **a** (scale bar: 2 μm). **c** Thickness distributions extracted from the AFM image (top) and the OM image (bottom); the *Y*-axes are probability density, namely, frequency per unit height; **d** Transmittance spectra of Ti_3_C_2_T_x_ films with various concentrations of injected Ti_3_C_2_T_x_; **e** Transmittance at 550 nm as a function of sheet resistance for the Ti_3_C_2_T_x_ films and data from previously reported transparent electrode technologies; RGO reduced graphene oxide, LB Langmuir–Blodgett. **f** Photograph of a Ti_3_C_2_Tx film deposited on a PET substrate that presents excellent transparency and flexibility.

To obtain large-area homogeneous thin films of the TiO_2_ or Ti_3_C_2_T_x_ network, a liquid/liquid interfacial self-assembly (LLIA) technique is used^28^. Specifically, the 2D flakes/IPA dispersion is injected to the interface between hexane and water. Because of the fast dissolution of IPA into the hexane layer, a surface tension gradient is established along the interface (Marangoni force)^29^. Driving by this Marangoni force, the 2D flakes spreads out quickly and settles at the edge of the liquid interface, that is, either the sidewall of the container or the edge of an already assembled film. By optimizing the amount of the injected 2D flakes/IPA solution, a full coverage is achieved and a continuous 2D thin film is obtained. As shown in the OM image of Fig. 3a, the LLIA Ti_3_C_2_T_x_ film is homogeneous over a large area. According to the AFM image (Fig. 3b and Supplementary Fig. 7) taken at the labeled area in Fig. 3a, the Ti_3_C_2_T_x_ flakes are all horizontally oriented, and placed next to each other with very small overlaps. This is very different from the films produced by the spray coating or spin coating methods where the orientations of the flakes are random, and they are mostly overlapped and bundled^30,31^. To quantify the uniformity of the LLIA film in a large area, we extract the height profiles (Supplementary Fig. 7) from the AFM image, use it to calibrate the optical contrast of the OM image to obtain the thickness distribution over the area (Supplementary Fig. 8). The thickness distributions extracted from both the AFM image (small area) and the OM image (large area) are shown in Fig. 3c. It is observed that the peaks for both distributions appear at around 2 nm, which corresponds to the thickness of 1-layer of Ti_3_C_2_T_x_ flake. The second most significant peak is at around 3.8 nm, which corresponds to the overlapped area of two adjacent flakes (2 layers). These 1-layer and 2-layer regions are the key functional components for the electrical conductance, which covers 64% and 18% of the film, respectively.

Because of the uniform thickness distributions and the high coverage of 1-layer and 2-layer, the LLIA Ti_3_C_2_T_x_ films show both high optical transparency and high electrical conductance, making them ideal for the transparent electrodes of EC devices. The optical and electronic properties are evaluated by ultraviolet-visible (UV-vis) spectrophotometry and a four-point probe resistance measurement technique. The UV-vis spectra of these films with different Ti_3_C_2_T_x_ area concentrations are presented in Fig. 3d, where the area concentration is defined as the mass of Ti_3_C_2_T_x_ per square centimeter, and tuned by the volume of 2 mg/ml Ti_3_C_2_T_x_ dispersion injected at the interface during the assembly. The transmittance of LLIA Ti_3_C_2_T_x_ at 550 nm reaches 96.8% when the area concentration is 1 µg cm^−2^, and decreases steadily to 64.3% as the area concentration increases to 60 µg cm^−2^ ^32^. The transmittance at 550 nm versus the sheet resistance (*R*_s_) of the LLIA films, together with the data from previously reported techniques, are plotted in Fig. 3e. The *R*_s_ value of the LLIA Ti_3_C_2_T_x_ film reaches 1623 Ω sq^−1^ at a transmittance of 96.9%, 210 Ω sq^−1^ at 94.1%, and 123 Ω sq^−1^ at 87.8%. Comparing the resistances of materials with the similar transparency, our values are lower than MXene films made by other methods like spin coating (201 Ω sq^−1^ at 87%), dip coating (4300 Ω sq^−1^ at 94%), and spray coating (7974 Ω sq^−1^ at 82%), and also lower than other transparent conductive materials such as graphene, and reduced graphene oxide (see Supplementary Table 1 for a summary of different transparent conductive materials)^30,31,33–40^. Finally, Fig. 3f shows that the LLIA Ti_3_C_2_T_x_ film can be transferred onto a flexible substrate (e.g., PET) uniformly in a large area (~25 cm^2^) with excellent optical transparency, suitable for transparent conductive electrodes for the proposed flexible EC devices.

### Fast and highly efficient electrochromism in MXene/TiO_2_ heterostructures

**Fig. 4:**
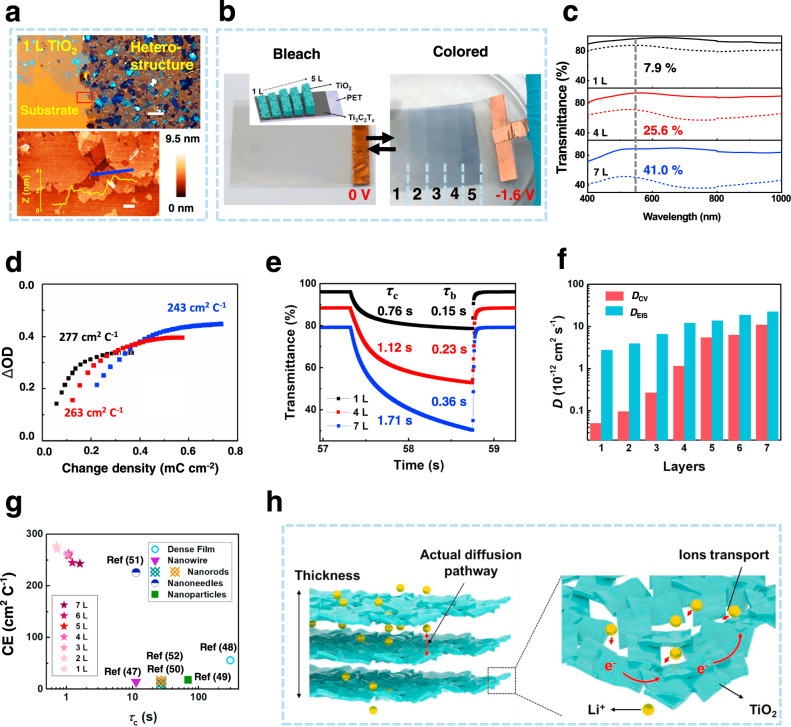
Electrochromic properties of the TiO_2_/Ti_3_C_2_T_x_ heterostructure. **a** OM image of a junction between a 1-layer TiO_2_ film and the TiO_2_/Ti_3_C_2_T_x_ heterostructure (Top) (Scale bar: 10 μm), and AFM image and the height profile of across the junction in corresponding area marked in the OM image (Bottom) (Scale bar: 1 μm); **b** Photographs of the TiO_2_/Ti_3_C_2_T_x_/PET film with different layers of TiO_2_ at the bleaching state (no bias) and the colored state (biased at −1.6 V). Inset: structure of TiO_2_/Ti_3_C_2_T_x_/PET film with different TiO_2_ layers; **c** Optical transmittance spectra with 1, 4, and 7 layers of TiO_2_ at the biases of 0 V (solid) and −1.6 V (dotted); **d** Optical density change (ΔOD) as a function of the injected charge density for the heterostructures with 1, 4, and 7 layers of TiO_2_; **e** Temporal response of the transmittance at 550 nm of the heterostructures with 1, 4, and 7 layers of TiO_2_; **f** Diffusion coefficients (*D*) of the heterostructures with 1−7 layers of TiO_2_ extracted from the EIS and CV measurements; **g** Coloration efficiency (CE) as a function of coloration time (*τ*_c_) for the TiO_2_/Ti_3_C_2_T_x_ heterostructures and previously reported TiO_2_-based structures; **h** Schematic of the pathways of electron conduction and ion diffusion within the self-assembled TiO_2_ thin film.

The flexible TiO_2_/Ti_3_C_2_T_x_ heterostructure electrodes are fabricated by transferring LLIA TiO_2_ films on the Ti_3_C_2_T_x_/PET substrates. Similar to Ti_3_C_2_T_x_, TiO_2_ flakes in the LLIA film also connect with each other to form a porous network, with the pore size in the range of nanometers, when observed under TEM and OM measurements (Supplementary Fig. 9 and Supplementary Fig. 10). The morphology of TiO_2_/Ti_3_C_2_T_x_ heterostructures are further characterized by OM, SEM, and AFM measurements. As shown in Fig. 4a, the boundary between the TiO_2_/Ti_3_C_2_T_x_ heterostructure and l-layer TiO_2_ film is clearly seen from the OM image. AFM characterization shows a thickness different of ~2 nm across the boundary, matching well with the thickness of 1-layer Ti_3_C_2_T_x_, indicating a close contact between the TiO_2_ and Ti_3_C_2_T_x_ layers. Moreover, Supplementary Fig. 11 shows OM and SEM images of different layers (1, 4, and 7 L) of LLIA TiO_2_ on Ti_3_C_2_T_x_ films, where the porous structures can be still seen in the stacked multiple layers.

The heterostructure provides a metal-semiconductor junction between Ti_3_C_2_T_x_ and TiO_2_, where Ti_3_C_2_T_x_ serves as the transparent electrode and TiO_2_ serves as the electrochromic layer. Note that Ti_3_C_2_T_x_ has a low work function (1.6–4.4 eV)^41,42^, when comparing to other transparent electrodes, e.g. indium tin oxide (ITO, with the work function of 4.73 eV)^43^. When contacting with TiO_2_ (work function ~4.2 eV), the Schottky barrier between Ti_3_C_2_T_x_ and TiO_2_ is much lower or it could even be an Ohmic contact^44^, compared to that between ITO and TiO_2_. Thus, the electron transport between the Ti_3_C_2_T_x_ and the TiO_2_ layer is expected to be greatly improved in the heterostructure.

**Fig. 5:**
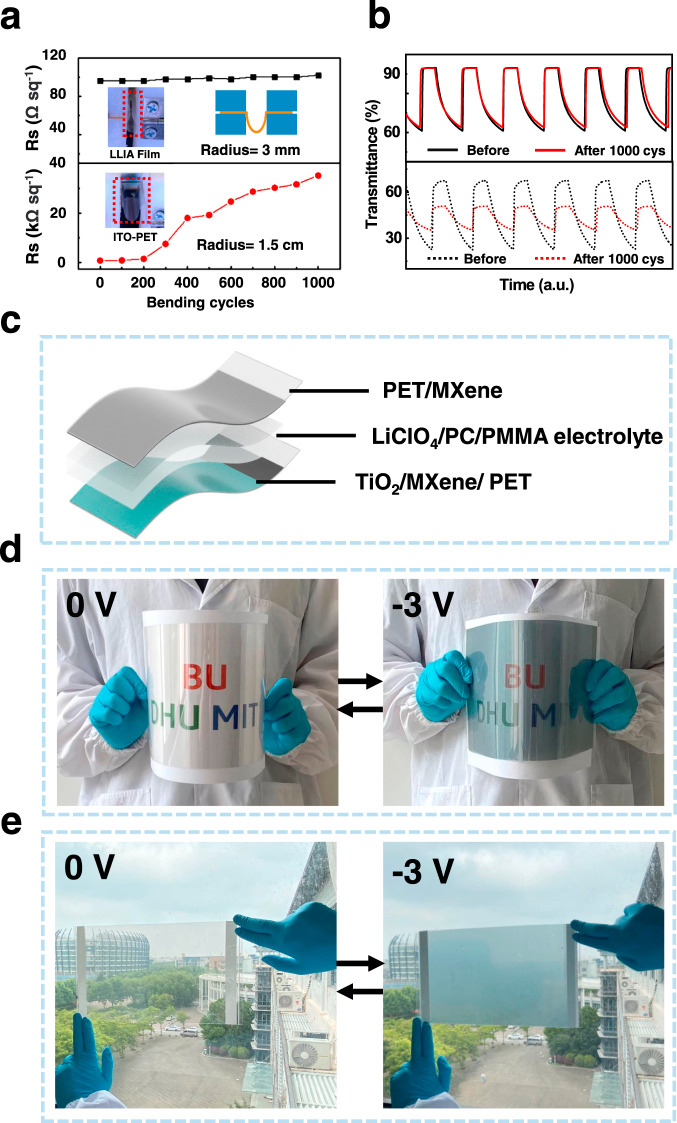
Mechanical properties of the TiO_2_/Ti_3_C_2_Tx heterostructure and demonstration of a large-area flexible EC device. **a** Comparison of the sheet resistance (*R*_s_) of the LLIA Ti_3_C_2_T_x_/PET and the ITO/PET films after 1000 bending cycles; **b** Comparison of the electrochromic switching behaviors of the LLIA TiO_2_/Ti_3_C_2_T_x_/PET and the ITO/PET films after 1000 bending cycles; **c** Schematic diagram of the flexible solid electrochromic device; **d**, **e** As-prepared solid electrochromic device with an A4 paper size (about 20 × 30 cm) is **d** triggered in bending state and **e** attached on a window.

Because of the well-controlled LLIA process, it is possible to regulate the EC properties simply by the thickness of the TiO_2_ layer. As shown in Figs. 4b, 1–5 layers of the LLIA TiO_2_ film are transferred sequentially onto a PET substrate in a layer-by-layer fashion. They are almost indistinguishable at the bleach state, when no bias is applied, but become translucent with different opacity at the colored state, when emerged in an electrolyte solution (1 M LiClO_4_/propylene carbonate (PC)) and biased with a constant negative voltage (−1.6 V). This optical opacity induced by a negative potential is associated with the reduction of TiO_2_ by Li^+^ counterions and the formation of Li_x_TiO_2_^45^.

As the in situ UV-Vis-NIR spectroscopy measurements shown (Fig. 4c and Supplementary Fig. 12), the optical modulation range Δ*T* changes with the increase of the number of layers of the transferred LLIA TiO_2_ film, where Δ*T is* defined as Δ*T* = *T*_b_ − *T*_c_ = exp(−*α*_b_*L*) − exp(−*α*_c_*L*) (*T*_b_ and *T*_c_ are the transmittance of the bleach state and the colored state, α_b_ and α_c_ represent absorption coefficient of the bleach and the colored state of the LLIA film, and L is the thickness of the film). Particularly, Δ*T* at the wavelength of 550 nm increases gradually with the layer numbers of the transferred LLIA TiO_2_ films (7.19%, 12.26%, 21.12%, 25.60%, 32.12%, 38.20%, and 41.09% from 1 to 7 layers) (Supplementary Table 2). Δ*T* reaches its maximum at around 60% with 15 layers of LLIA TiO_2_ films and then declines as the layers number reaches to 20. This is because the transmittance of the bleach state decreases significantly when the film becomes thick, resulting in the decrease of the optical window (Supplementary Fig. 13). We further extracted Δ*α* = *α*_c_ – *α*_b_ ≈ [ln(*T*_c_/*T*_b_)]/L from the transmittance spectra. Compared to the TiO_2_-based EC film prepared using other methods (Supplementary Table 3)^16,19,46–59^, our LLIA TiO_2_ film shows a Δ*α* of 0.12 nm^−1^, which is about one order of magnitude higher. This results suggest that more TiO_2_ per unit volume in our LLIA TiO_2_ film participate in the EC process. Such high utilization rate of TiO_2_ in the LLIA films is one of the key factors responsible for the improved EC performance.

To quantify the utilization rates of both the EC material and the pumped charge, the optical density change (ΔOD = log_10_(*T*_b_/*T*)) as the function of the pumped charge density (*Q*, calculated by an integration of the driving current density over time) during the coloration half-cycle is measured and plotted in Fig. 4d. The coloration efficiencies (CE)^60^, defined as the slope of the ΔOD-*Q* plot when *Q* is close to 0, for the LLIA TiO_2_ films with 1–7 layer numbers are extracted to be 277, 271, 260, 263, 259, 245, and 243 cm^2^ C^−1^, respectively **(**Fig. 4d and Supplementary Table 2). These high CE values indicate that small amounts of charge insertion/extraction can result in a large optical modulation (normally CE values of TMO-based EC materials are lower than 100 cm^2^ C^−1^), which is a results of high utilization rates of both the EC materials and the pumped charges. High CE also guarantees the long-term electrochemical stability. As expected, the LLIA films show maintained optical contrast (up to 95% of its original value) after 500 EC cycles (Supplementary Fig. 14 and Supplementary Fig. 15). This high cycling stability is essential for practical applications of the EC devices.

The coloration/bleaching time *τ*_c_/*τ*_b_, another important figure-of-merit for EC materials, defined as the time needed for 90% of the total optical change, is also investigated on the LLIA TiO_2_/Ti_3_C_2_T_x_ heterostructures. The temporal responses of the transmittance at 550 nm are measured as the bias is switched between −1.6 and 0 V periodically (Fig. 4e). The coloration/bleaching times for 1–7 layers of the LLIC TiO_2_ films are extracted to be 0.76/0.15 s, 0.73/0.12 s, 1.03/0.20 s, 1.03/0.23 s, 1.08/0.26 s, 1.23/0.29 s, and 1.71/0.36 s (also summarized in Supplementary Table 2), which are much shorter than those of reported TiO_2_-based EC devices (generally >10 s) and most reported inorganic materials, and comparable to those of organic EC materials (Supplementary Table 3).

Ion diffusion process inside the EC material plays a crucial role in the EC performance. To further explore the Li^+^ diffusion behavior in the LLIA TiO_2_ films during the EC process, cyclic voltammetry (CV) and electrochemical impedance spectroscopy (EIS) are measured (details are listed in the Method section). All LLIA films exhibit one pair of redox peaks in their CV curves, corresponding to the reduction and oxidation of TiO_2_ (Supplementary Fig. 16). According to the EIS results shown in Supplementary Fig. 17 and Supplementary Table 4, the equivalent series resistance (ESR) does not change much as the TiO_2_ thickness increases from 1 to 7 layers, and the charge-transfer resistance (*R*_ct_) only raises from 8.7 to 15.7 Ω, indicating that multilayer stacking does not decrease the conductivity and charge-transfer speed much at the interface of the TiO_2_ film and electrolyte.

The apparent ion diffusion coefficient *D*, which represents the kinetics of ion insertion/extraction in the EC film, can be extracted either from the scanning-rate-dependent CV measurements, or from the Warburg region of the Bode plot obtained from the EIS measurements (see methods for details about the extraction methods of *D*)^61^. As shown in Fig. 4f, the *D* values extracted from the CV and from the EIS measurements are 5.05 × 10^−14^, 9.63 × 10^−14^, 2.68 × 10^−13^, 1.15 × 10^−12^, 5.45 × 10^−12^, 6.29 × 10^−12^, and 1.10 × 10^−11^ cm^2^ s^−1^, and 2.75 × 10^−12^, 3.94 × 10^−12^, 6.59 × 10^−12^, 1.21 × 10^−11^, 1.39 × 10^−11^, 1.88 × 10^−11^, 2.24 × 10^−11^ cm^2^ s^−1^, respectively, as the number of layer of the TiO_2_ film increases from 1 to 7. The positive correlation between *D* and the layer number is attributed to the increased effective electrochemical area of the LLIA TiO_2_ film due to its high surface-to-volume ratio, which will be explained later.

To benchmark the overall EC performance, the values of *CE* and *τ*_c_ of the LLIA TiO_2_/Ti_3_C_2_T_x_ heterostructures as well as previously reported EC materials are summarized in Fig. 4g and Supplementary Table 3. It is clearly observed that both the *CE* and the response time of our proposed structures are several times or even one order of magnitude better than those from both other materials such as WO_3_, and other nanostructured TiO_2_, including dense films and other 0D or 1D nanostructures, and comparable to organic materials. Moreover, the good cycling stability of our LLIA TiO_2_/Ti_3_C_2_T_x_ heterostructures could address the ultraviolet/electrochemical decomposition issues in traditional organic materials in practical applications^18^.

We believe that the superiority of the EC performance of the LLIA TiO_2_/Ti_3_C_2_T_x_ heterostructures mainly results from the 2D nature of the TiO_2_ flakes as well as the loosely networked structures. To understand this, we propose two possible ion transport pathways^15^. The first ion transport pathway is that the Li^+^ ions enters the EC material only on the top surface, and the redoxation deeper into the EC material is accessed by inter-crystal-lattice diffusion of Li^+^ ions; whereas in the second ion transport pathway (as illustrated in Fig. 4h), Li^+^ ions could travel to all the surfaces of individual EC nanostructures efficiently in the liquid phase, and as a result, the diffusion and the redoxation take place throughout the entire EC nanostructures simultaneously. For dense films, only the first pathway takes place, resulting in very bad coloration efficiency and very slow coloration/bleaching time; while for nanostructured films (including our 2D nanostructures and previously reported 0D or 1D nanostructures), the second pathway, which is much more efficient and much faster, starts to play an important role. The higher probability the second pathway takes place in a nanostructured film, the better EC performance it can achieve.

Our experimental results suggest that the second pathway is the dominating Li^+^ ion transport mechanism in our self-assembled 2D TiO_2_ films. First, the high *CE* and its small decrease with the thickness suggest that Li^+^ ions can easily access all the TiO_2_ flakes regardless of the depth of them from the top surface of the film. Second, the color change time *τ* (*τ*_c_ or *τ*_b_) is fast and stays almost the same with different thicknesses. Given that *τ* is proportional to the square of the depth of the diffusion, we speculate that the effective diffusion depth is likely to be determined by the average thickness of individual TiO_2_ flakes, rather than the thickness of the entire assembled TiO_2_ film. Last, the increasing trend between *D* and the thickness indicates that it is less likely that the extremely slow internanosheet diffusion is contributed to the measured apparent diffusion coefficient. Instead, *D* follows a parabolic trend with the thickness (see Supplementary Fig. 18), or the number of layers, which agrees well if considering the contribution from the electrochemical area *A*_EC_ and assuming a linear increase of *A*_EC_ with the number of layers, that is, *D*~(*A*_EC_/*A*)^2^*D*_0_ ∝ *N*^2^, where *A* is the geometrical area, *D*_0_ is the intrinsic diffusion coefficient, and *N* is the number of layers. All these experimental observations can be explained well if the performance is governed by the second ion transport pathway, in which all the surfaces of the 2D TiO_2_ flakes are interfacing well with the electrolyte, and the actual diffusion length is approximately the thickness of individual TiO_2_ flakes (Fig. 4h). As a comparison, for 1D or 0D nanostructures, although the ideal surface-to-volume might be even larger, the surfaces may not be fully exposed to the electrolyte because of the denser packing of these nanostructures in a film. Consequently, the Li^+^ ions have to undergo the first pathway in order to access the deeper EC materials, which tremendously limits the speed and thus reduces the coloration efficiency.

### MXene/TiO_2_ heterostructure enabled flexible EC devices for large-area applications

The overall flexibility of an EC device is determined by the most rigid layer. In our TiO_2_/Ti_3_C_2_T_x_ heterostructure, both the EC layer and the transparent electrode have superior mechanical bendability. To confirm this, bending test is performed on the as-fabricated LLIA electrode. As shown in Fig. 5a, the resistance of the Ti_3_C_2_T_x_/PET film increases by only 6% after 1000 bending and release cycles at a bending radius of 0.3 cm, whereas the sheet resistance of a commercial ITO/PET film increases quickly by three orders of magnitude after 1000 bending cycles even at a much larger bending radius of 1.5 cm. The EC optical switching behaviors of the TiO_2_/Ti_3_C_2_T_x_/PET film and the TiO_2_/ITO/PET before and after 1000 bending cycles are also tested (Fig. 5b). Δ*T* of the TiO_2_/Ti_3_C_2_T_x_/PET electrode remains at 94% of its original value after 1000 bending cycles. In contrast, Δ*T* of the TiO_2_/ITO/PET electrode drops to about 30% after 1000 cycles.

Finally, a large-area flexible EC device with a dimension of 30 × 20 cm are demonstrated as an example for practical large-area applications. As shown in Fig. 5c, it is composed of a LLIA TiO_2_/Ti_3_C_2_T_x_ heterostructure on a PET substrate as the EC electrode, another LLIA Ti_3_C_2_T_x_/PET film as the counter electrode, and a 1 M LiClO_4_/PC/Poly(methyl methacrylate) (PMMA) layer as the electrolyte. Benefiting from the exceptional optical memory effect of TiO_2_ based materials, the EC device can maintain its coloration state after disconnecting from the voltage supply (Fig. 5d, e), which is advantageous for energy saving in future applications. The EC device presents a uniform color distribution even in the bending state, which opens up the possibility of scaling up this technology for applications, such as flexible displays, smart windows, and other ubiquitous electronics. In addition, the flexible solid EC device also exhibits excellent cycling stability, demonstrated by the 92% transmittance presentation after 1000 EC cycles between the bias of -3 V and 1 V (**S**).

## Discussion

In summary, high performance and flexible EC devices are realized based on assembled 2D TiO_2_/Ti_3_C_2_T_x_ heterostructures. This success benefits from several important achievements in this work, including the realization of single crystalline 2D TiO_2_ flakes with micrometer large lateral sizes and nanometer thin thicknesses, and the liquid/liquid interfacial self-assembly technique enabled mass production of MXene and 2D TMO films with excellent uniformity. We expect our approach to synthesize 2D TiO_2_ crystals to be developed to a general methodology for the synthesis of high-quality 2D MXene derivatives. Also, the self-assembled MXene films could be used as flexible, transparent, and porous electrodes in large-scale electronic, optoelectronic, and energy-harvesting applications. Our performance test clearly shows that exceptional electrochromic efficiency, fast coloration speed, and excellent mechanical flexibility are achieved simultaneously on the self-assembled 2D TMO/MXene heterostructures-based EC devices, thanks to the unique electrical, electrochemical, and mechanical properties of the networked 2D nanostructures. Finally, a large-area flexible EC device is demonstrated, paving the way for integrating EC devices to next-generation flexible and wearable electronics.

## Methods

### Chemicals

All the starting materials are available commercially and used as received. Polyethylene terephthalate (PET) was rinsed sequentially in the baths of deionized water, acetone, and isopropanol. Ti_3_AlC_2_ (MAX-Phase, purchased from 11 Technology Co. Ltd), lithium fluoride (LiF, Alfa Aesar, 99.99%), hydrochloric acid (HCl, Fisher Chemical, 6M), 1-hexene (Acros Organics, 99%), isopropyl alcohol (IPA, Fisher Chemical, 99%), propylene carbonate (PC, Alfa Aesar, 99%), lithium perchlorate (LiClO_4_, Acros Organics, 99+%), and poly(methyl methacrylate) (PMMA, ACROS Organics, M.W. 35000) were utilized without initial treatments.

### Synthesis and interfacial assembly of Ti_3_C_2_T_x_ and 2D TiO_2_ films

Ti_3_AlC_2_ etching was performed following Gogosti et al.’s work^22^ to prepare 2D Ti_3_C_2_T_x_. Briefly, 1.6 g lithium fluoride was dissolved into 5 mL of DI water with 15 mL 12 M hydrochloric acid solution in a plastic bottle as the etchant solution. Then 1 g Ti_3_AlC_2_ (MAX phase) powders was gradually added to the etchant solution within 5 min and the reaction is maintained with stirring the reactants at 40 °C in a silicon oil bath for 24 h. Etched MXene was washed with DI water, and separated through centrifugation at 1988 × *g* for 5 min. Washing was repeated several times until a stable dark supernatant solution of Ti_3_C_2_T_x_ flakes with a pH value of around 6 was obtained. The solution was subsequently centrifuged for 30 min at 3500 rpm, after which a dark sediment of Ti_3_C_2_T_x_ was observed and then collected.

The Ti_3_C_2_T_x_ sedimen was diluted to 0.2 mg/ml with DI water, and then, the solution were freeze dried at −47 °C for days to form an aerogel. To obtaine the TiO_2_ aerogel, the Ti_3_C_2_T_x_ aerogel was put in a ceramic boat and annealed at the temperature of 400 °C for 50 h with the heating rate of 1 °C/min.

To assemble the Ti_3_C_2_T_x_ flakes into a continuous film, the Ti_3_C_2_T_x_ aerogel was dispersed in an IPA solution (2 mg/ml) by sonicating for 30 min. Hexane was carefully poured on the top of DI water to setup an interface between these two liquids. Then, the Ti_3_C_2_T_x_/IPA dispersion was slowly injected near the interface to form a continuous film. After removing the top hexane layer, the self-assembly film was transferred onto a substrate and annealed at 90 °C in vacuum for 50 h (<0.05 MPa).

The assembly of the TiO_2_ films is similar to the Ti_3_C_2_T_x_ assembly process. For multilayered TiO_2_ film, the transferred films were dried at 60 °C in vacuum for 1 h, and then treated with oxygen plasma (the oxygen gas flow rate is 50 sccm, and the plasma power is 100 W) for 30 s before each transfer cycle. The TiO_2_/Ti_3_C_2_T_x_ heterostructures were annealed at 90 °C in vacuum (<0.05 MPa) for 50 h before use.

### Assembly of the Electrochromic device

The electrolyte gel was obtained by mixing 1 M LiClO_4_ in a PC solution and 20 wt% PMMA (relative to the LiClO_4_/PC solution). For the large-area (20 × 30 cm) electrode, silver paste line is screen printed around the Ti_3_C_2_T_x_/PET film and then protected by continuous printed UV curing resin to improve the electrical conductivity. The EC devices were fabricated by hot pressing the electrolyte gel sandwiched betwen the TiO_2_/Ti_3_C_2_T_x_/PET electrode and the Ti_3_C_2_T_x_ /PET counter electrode with a scotch tape spacer (with the thickness of 1 mm) at 60 °C.

### Material characterization

X-ray diffraction (XRD) patterns were measured using an X-ray diffractometer (Bruker AXS X8 Proteum-R instrument) with the radiation at 40 kV and 200 mA. Raman spectroscopy measurements were performed on a Renishaw inVia Raman microscope equipped with a 532 nm laser line (The laser power on the samples is about 1.6 mW). All the spectra in comparison were taken under the same conditions. Transmission electron microscopy (TEM) measurements were performed on a FEI Tecnai Osiris TEM. Selected area electron diffraction (SAED) was measured on a JEOL 2100 TEM. AFM measurements were conducted on a Bruker Dimension system. X-ray photoelectron spectroscopy (XPS) measurements were performed using a PHI Versaprobe II. The UV-vis spectroscopy was carried out using a Agilent CARY 5000.

### Electrochemical measurements and parameter extraction

Electrochemical measurements were carried out in a PC solution containing 1 M LiClO_4_ electrolyte using three-electrode electrochemical cells with the TiO_2_/Ti_3_C_2_T_x_/PET films as the working electrodes, a platinum plate as the counter electrode, and a freshly prepared Ag/Ag^+^ as the pseudo-reference electrode (calibrated by ferrocene). Electrochemical impedance spectroscopy (EIS) and cycle voltammetry (CV) were conducted by an electrochemical workstation (Bio-logic SAS, VSP-300) over a frequency range of 100 mHz–1000 KHz.

The measured EIS spectra was fitted with a circuit model illustrated in u. The Nyquist plot of all films is composed of three parts: an equivalent series resistance (ESR) in series with a semicircle consisting of a non-faradaic double layer capacitance (*C*_dl_) and a charge-transfer resistance (*R*_ct_) at high frequency; a 45° linear region representing the Warburg element (*W*), which captures the ion diffusion process at medium frequency; and a line related to the capacitive charging behavior of the pseudo-capacitance (*C*_p_) of TiO_2_ film and its bulk resistance (*R*_p_) at low frequency.

The apparent ion diffusion coefficient *D* can be extracted from both the EIS measurements and the CV measurements. In the first method, *D*_EIS_ can be calculated from the Warburg region according to Eqs. (1) and (2):^61,62^

1 $$Z_w = \sigma \omega ^{-1/2}$$

2 $$D_{{\mathrm{EIS}}} = R^2T^2/(2A^2n^4F^4C^2\sigma ^2)$$

where *σ* is the slope of *Z*_w_ against *ω*^−1/2^, *R* is the gas constant, *T* is the absolute temperature, *A* is the effective working area (geometrical area), *n* is the number of electrons involved in the electrochemical reaction*, F* and *C* are the Faraday constant and the molar concentration of Li^+^ ions.

In the second method, the apparent ion diffusion coefficients *D*_CV_ can be calculated by:^63^

3 $$i_p = 2.687 \times 10^5 \times n^{3/2} \times D_{CV}^{1/2} \times C \times A \times v^{1/2}$$

where *v* represents the potential sweeping rate, *i*_p_ represents the peak current density.

## Supplementary information

Supplementary Information

Description of Additional Supplementary Files

Supplementary Movie 1

Supplementary Movie 2

## Data Availability

All data needed to evaluate the conclusions in the paper are present in the paper and/or the Supplementary Information. Additional data related to this paper may be requested from the authors.
