# Supplementary material for: Flexible and high-performance electrochromic devices enabled by self-assembled 2D TiO2/MXene heterostructures
Source: Nat Commun. 2021 Mar 11;12:1587. doi: 10.1038/s41467-021-21852-7 (PMC7952574; doi:10.1038/s41467-021-21852-7)
Supplement: Supplementary file 1 — Supplementary Information [file 41467_2021_21852_MOESM1_ESM.pdf]

Supplementary Materials

Flexible and High-Performance Electrochromic Devices Enabled by Self-Assembled 2D TiO_2/MXene Heterostructures

Ran Li[1,2]†, Xiaoyuan Ma[2]†, Jianmin Li[1], Jun Cao[2], Hongze Gao[3], Tianshu Li[3], Xiaoyu Zhang[1], Lichao Wang[1], Qinghong Zhang[1], Gang Wang[1], Chengyi Hou[1], Yaogang Li[1], Tomás Palacios[4], Yuxuan Lin[4*#], Hongzhi Wang[1*], and Xi Ling[2,3,5*]

[1]State Key Laboratory for Modification of Chemical Fibers and Polymer Materials, College of Materials Science and Engineering, Donghua University, Shanghai 201620, China.
[2]Department of Chemistry, Boston University, 590 Commonwealth Avenue, Boston, MA 02215, USA.
[3]Division of Materials Science and Engineering, Boston University, 15 St. Mary's street, Boston, MA 02215, USA.
[4]Department of Electrical Engineering and Computer Science, Massachusetts Institute of Technology, Cambridge, MA 02139, USA.
[5]The Photonics Center, Boston University, 8 St. Mary's street, Boston, MA 02215, USA.

†These authors contributed equally to this work.

[*]Correspondence to: (X.L.) xiling@bu.edu; (H.W.) wanghz@dhu.edu.cn; (Y.L.) yxlin@berkeley.edu

[#]Current address: Department of Electrical Engineering and Computer Sciences, University of California, Berkeley, CA 94720, USA.

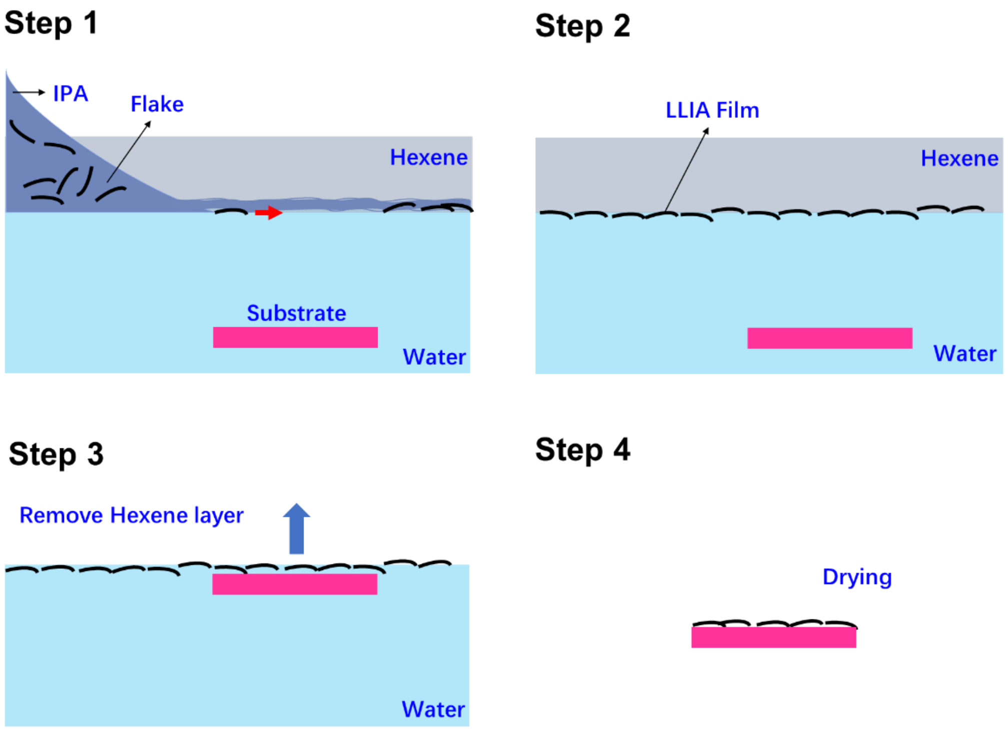

Supplementary Figure 1. Schematic illustration of the self-assemble process at the IPA/water interface and the transfer process.

Fig. S1 shows the LLIA assembly and film transfer processes: (1) Add 2D flakes/IPA dispersion into the hexane/water interface; (2) After the film formed at the interface, remove the hexane layer carefully; (3,4) Take substrate out from water and dry it in the oven (PET films are pasted on a glass substrate to keep it in shape when taking out from water). In this process, due to the density difference of the liquids, IPA solvent is quickly dropped to the bottom of the hexene layer and spread at the hexene/water interface at first. Then, flakes that are originally aggregated in the IPA phase spread out on the water surface. When the IPA mixture with hexene becomes thinner, the 2D flakes spread out quickly and settles at the edge of the container sidewall or an already assembled film. Continuous addition of the flakes eventually leads to the formation of a large-area monolayer of the material. The formation process of the films is recorded in the video S1 and video S2.

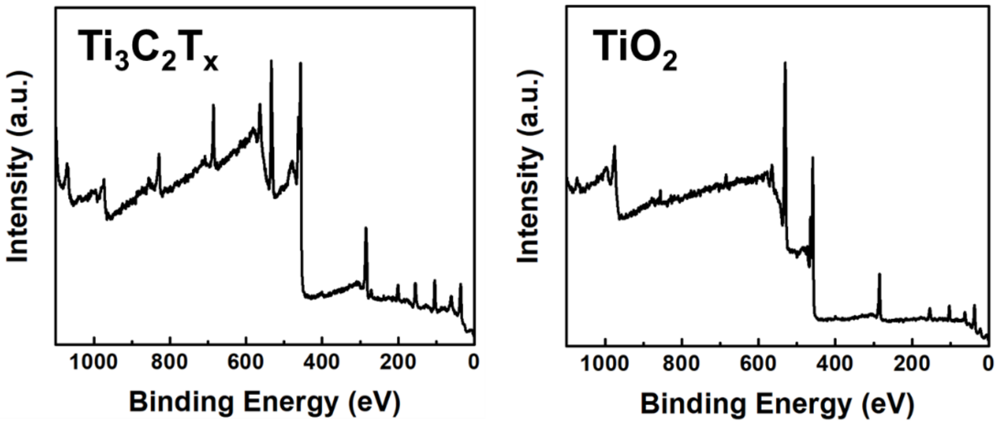

Supplementary Figure 2. XPS spectra of $Ti_3C_2T_x$ and derived TiO_2 annealed at 400°C for 50 h.

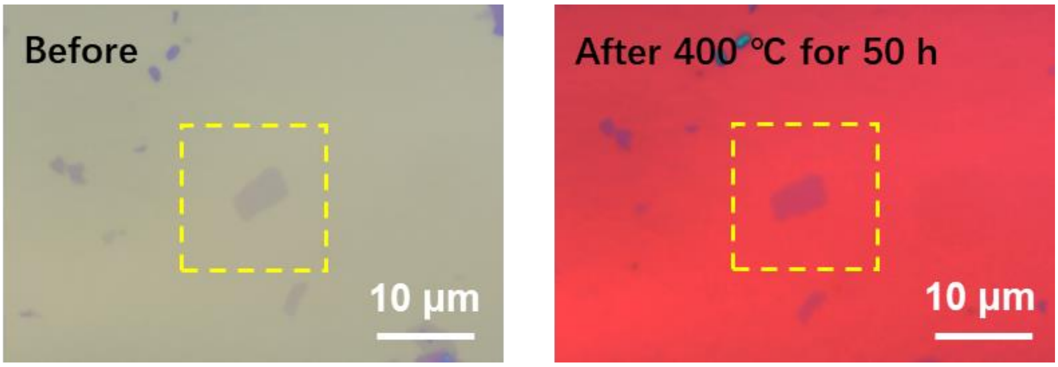

Supplementary Figure 3. $Ti_3C_2T_x$ flake before and after annealing at 400°C for 50 h on a SiO_2/Si substrate (the change of the background color is due to the interference effect caused by the change of the SiO_2 thickness after annealing).

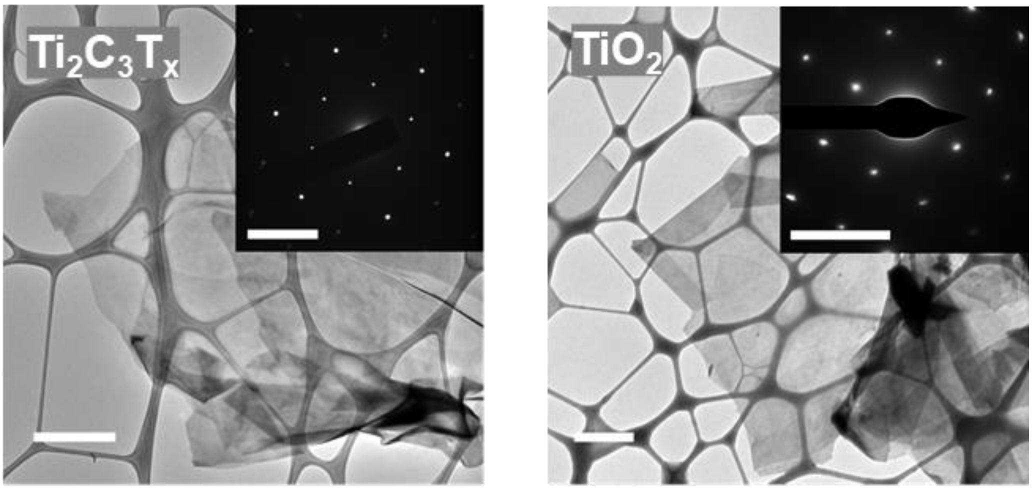

Supplementary Figure 4. TEM images of $Ti_3C_2T_x$ and derived TiO_2 flakes on a TEM grid (scale bar: 1 μm); Insets: SEAD patterns of $Ti_3C_2T_x$ and derived TiO_2 flakes (scale bar: 5 nm^{-1}).

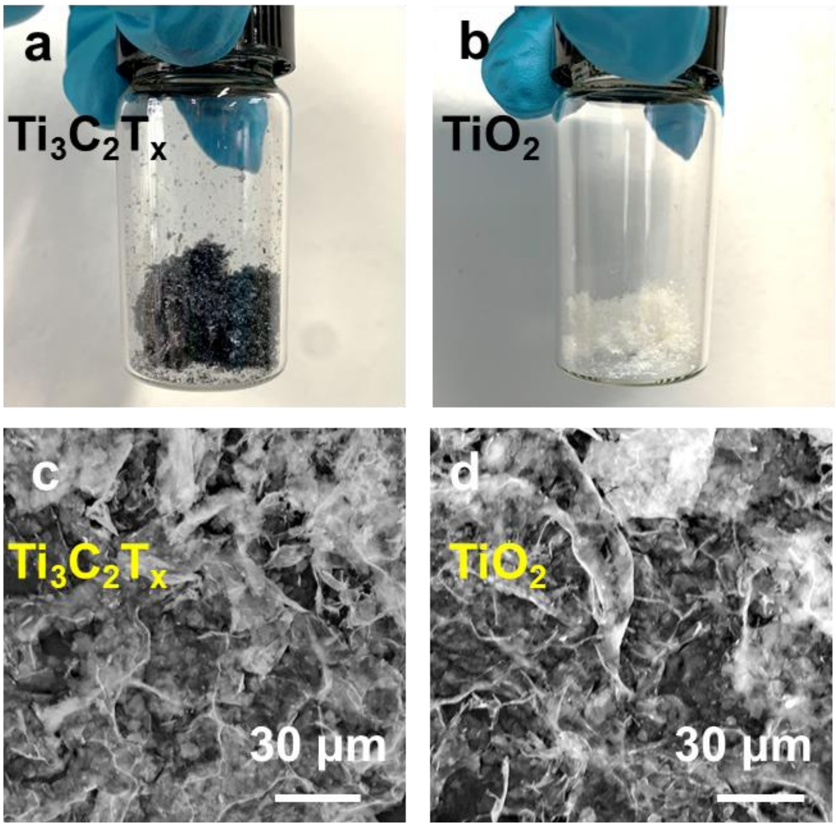

Supplementary Figure 5. Photographs of (a) $Ti_3C_2T_x$ and (b) TiO_2 aerogels; SEM images of (c) $Ti_3C_2T_x$ and (d) TiO_2 aerogels.

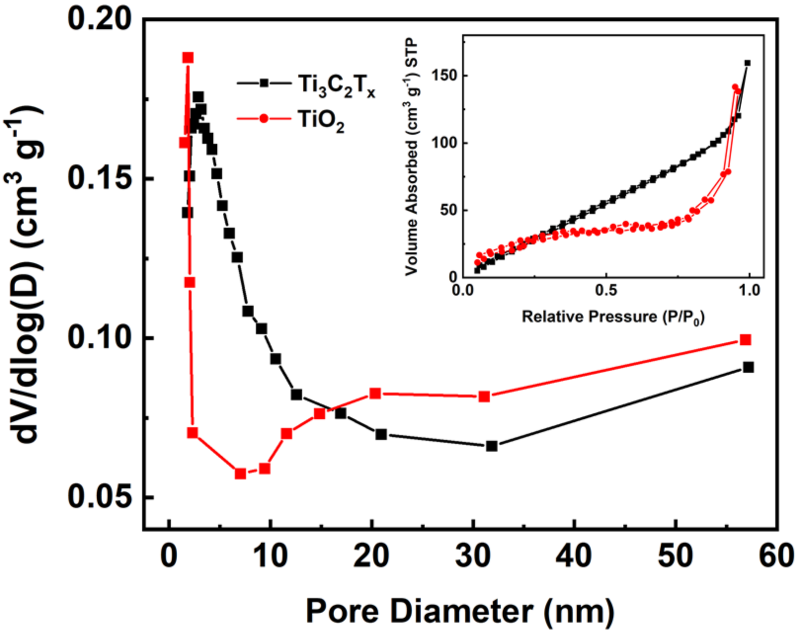

Supplementary Figure 6. Pore size distributions of $Ti_3C_2T_x$ (black) and TiO_2 (red) aerogels extracted using the Barrett-Joyner-Halenda method. Inset: N_2 adsorption and desorption isotherm curves for $Ti_3C_2T_x$ and TiO_2 aerogels measured at 77 K.

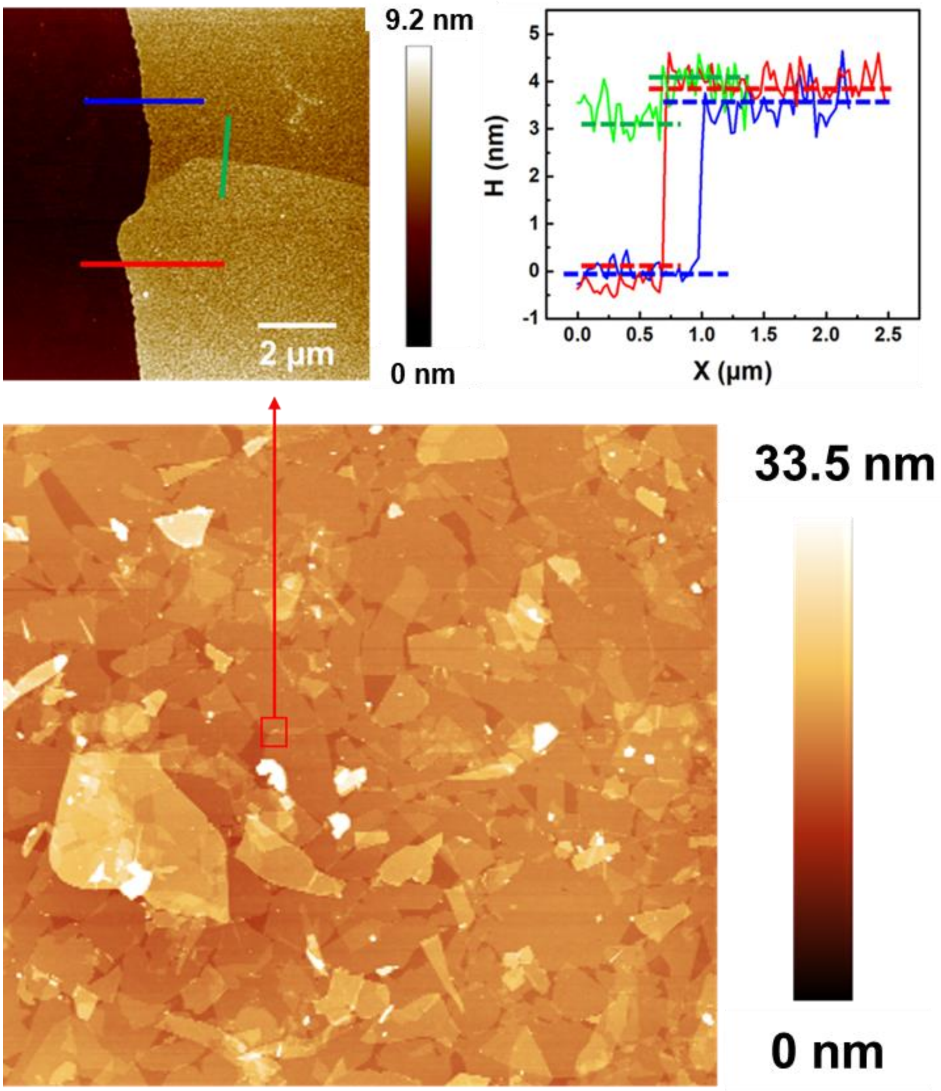

Supplementary Figure 7. AFM images and height profile of the LLIA $Ti_3C_2T_x$ thin film.

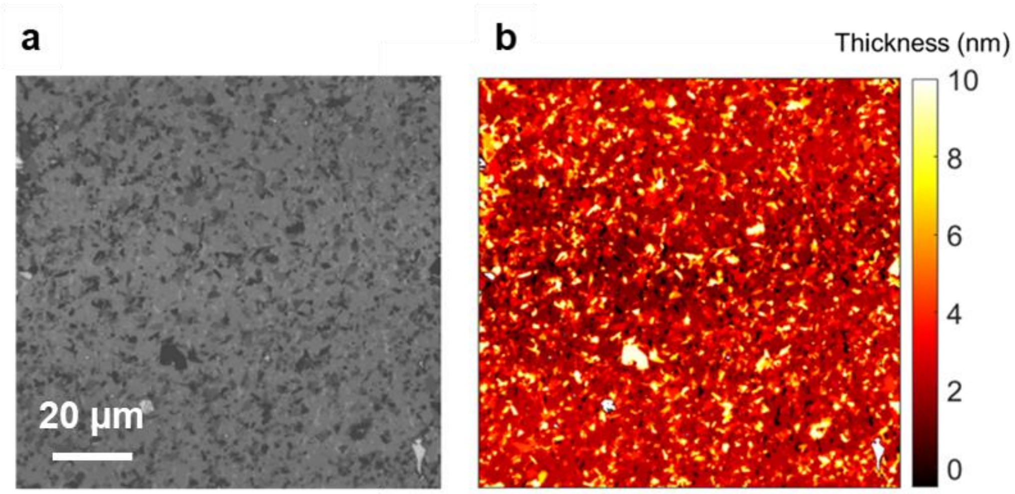

Supplementary Figure 8. (a) Optical microscope image (in gray scale) of the LLIA $Ti_3C_2T_x$ thin film; (b) height mapping obtained by calibrating (a) by the selective-area AFM image.

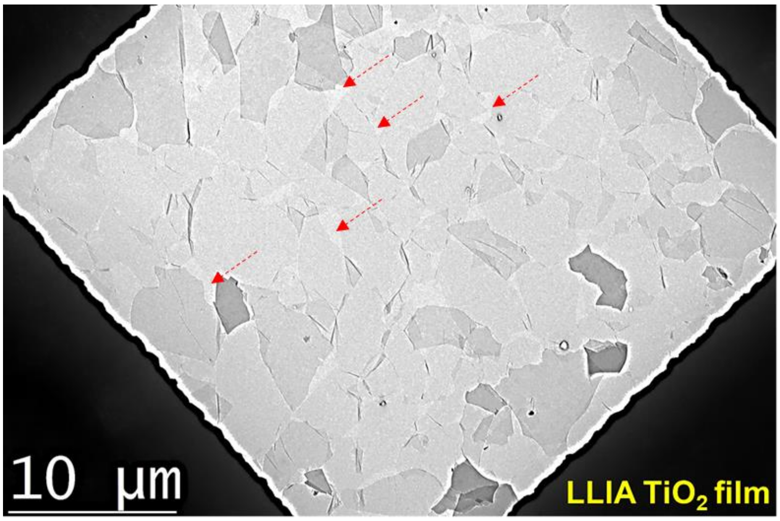

Supplementary Figure 9. TEM image of LLIA TiO_2 film on a TEM grid (typical pores are pointed by arrows).

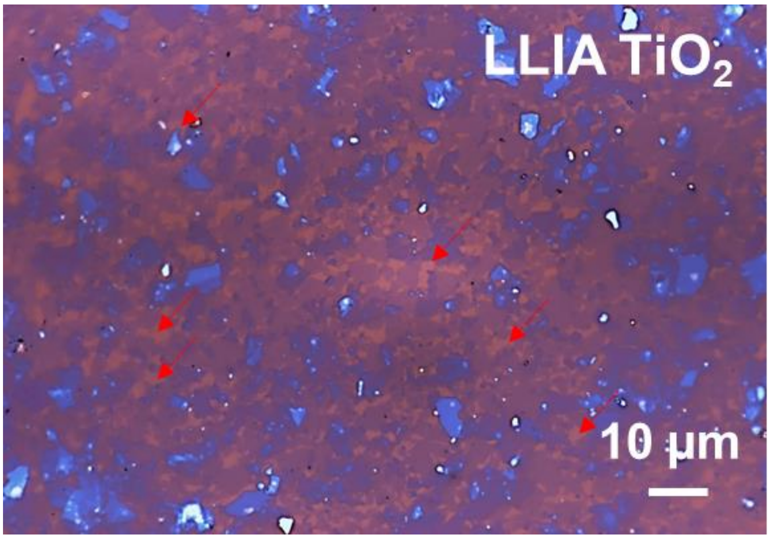

Supplementary Figure 10. OM image of LLIA TiO_2 film on a SiO_2/Si wafer (typical pores are pointed by arrows).

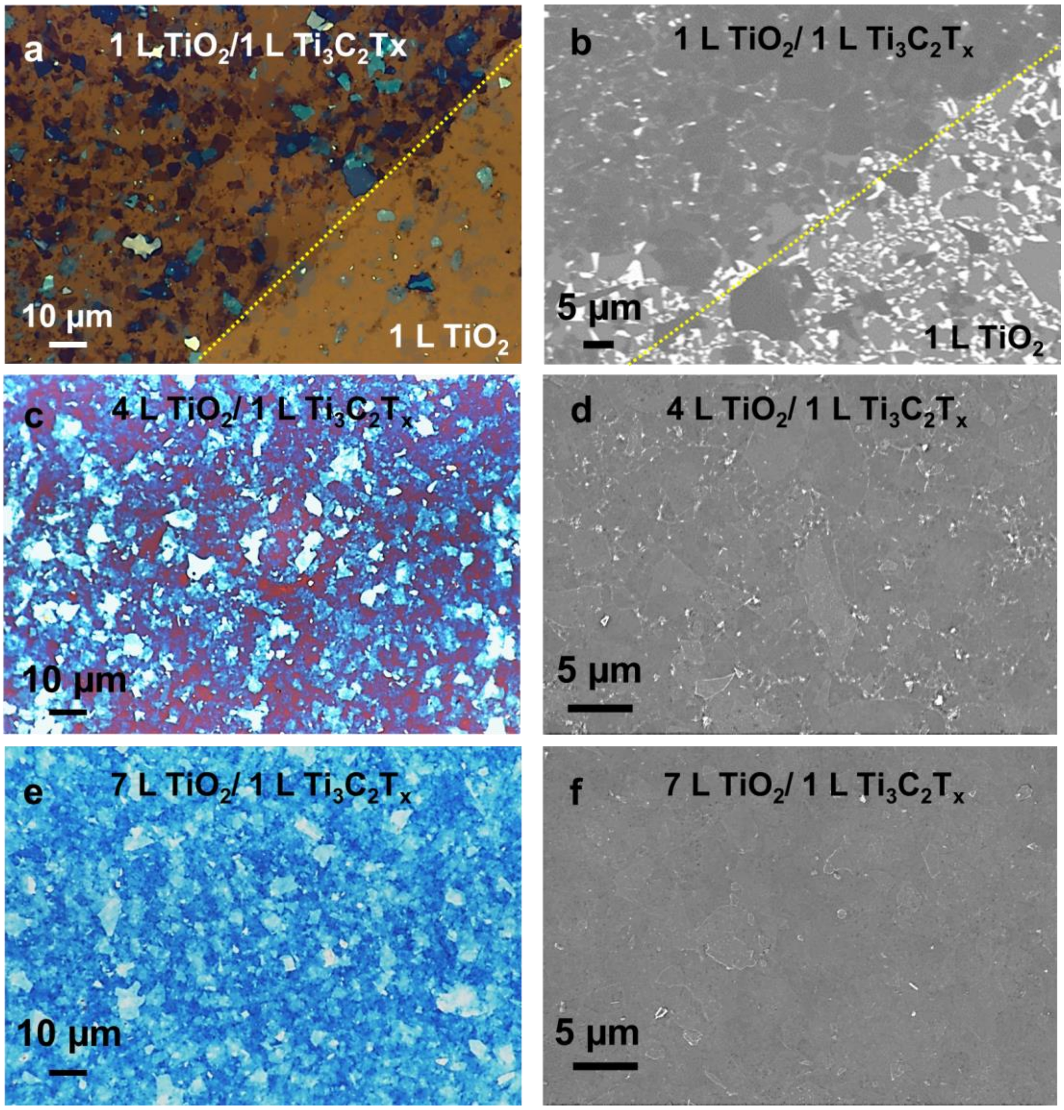

Supplementary Figure 11. OM images of assembled heterostructures with (a) 1-layer, (c) 4-layers and (e) 7-layers of TiO_2 on a 1-layer of $Ti_3C_2T_x$ on a SiO_2/Si substrate; SEM images (b, d and f) of the corresponding structures in (a-f).

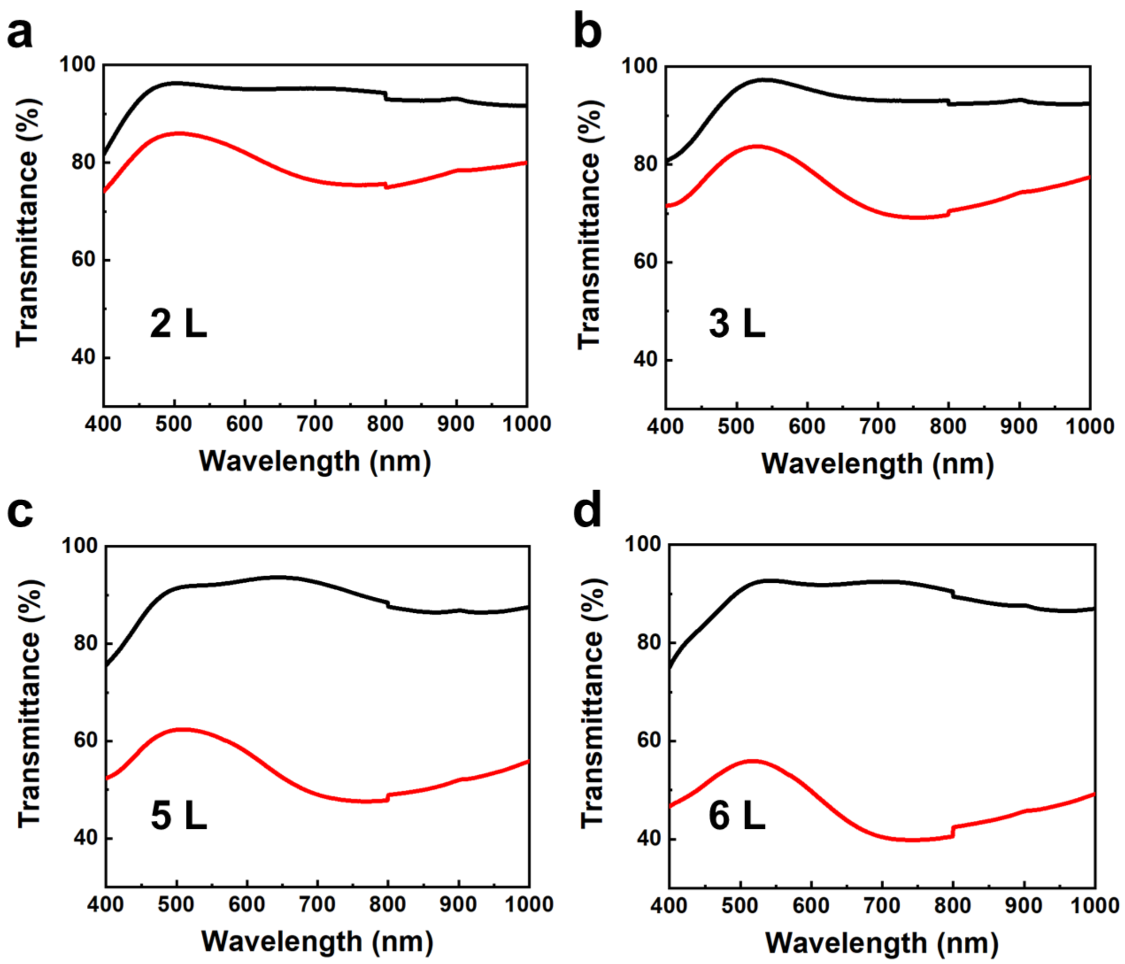

Supplementary Figure 12. Optical transmittance spectra of $TiO_2/Ti_3C_2T_x$/PET films with 2, 3, 5 and 6 layers of TiO_2 at the biases of 0 V (black) and -1.8 V (red).

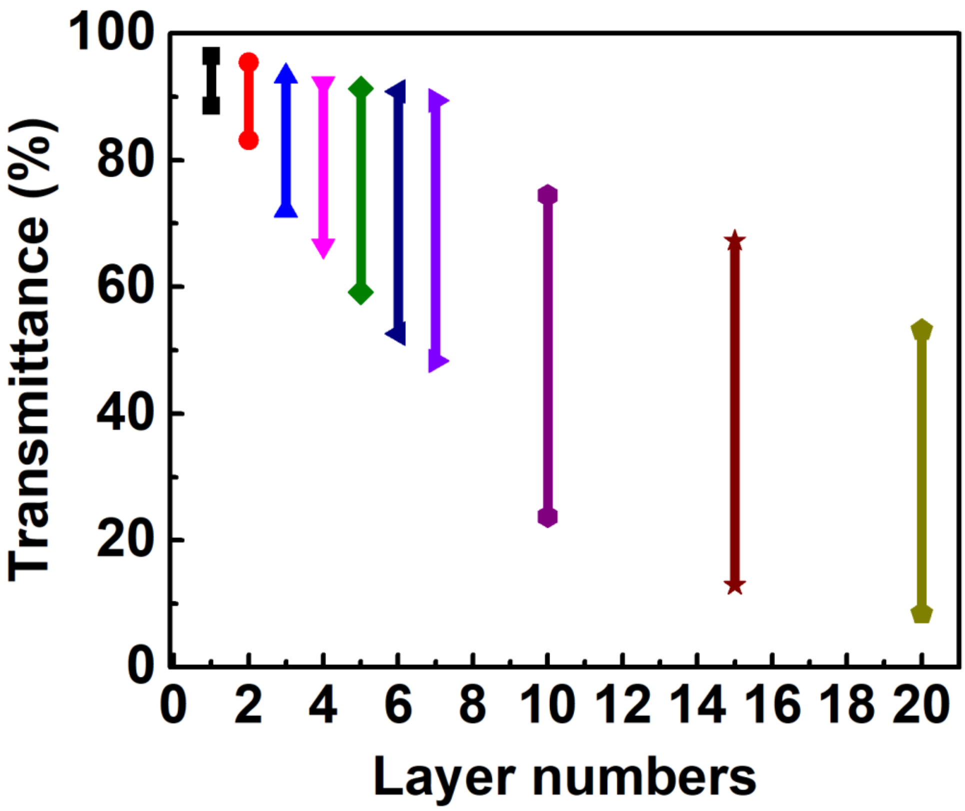

Supplementary Figure 13. Optical window of the heterostructures with different layers of LLIA TiO_2 films at the transmittance at 550 nm.

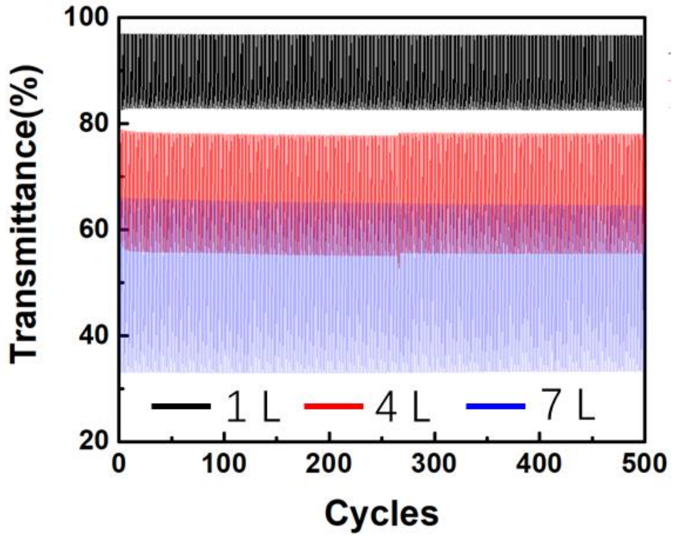

Supplementary Figure 14. Cycling stability of $TiO_2/Ti_3C_2T_x$/PET films with 1, 4, and 7 layers of TiO_2 with the transmittance measured at 550 nm and the voltage changed periodically between 0 V and -1.6 V.

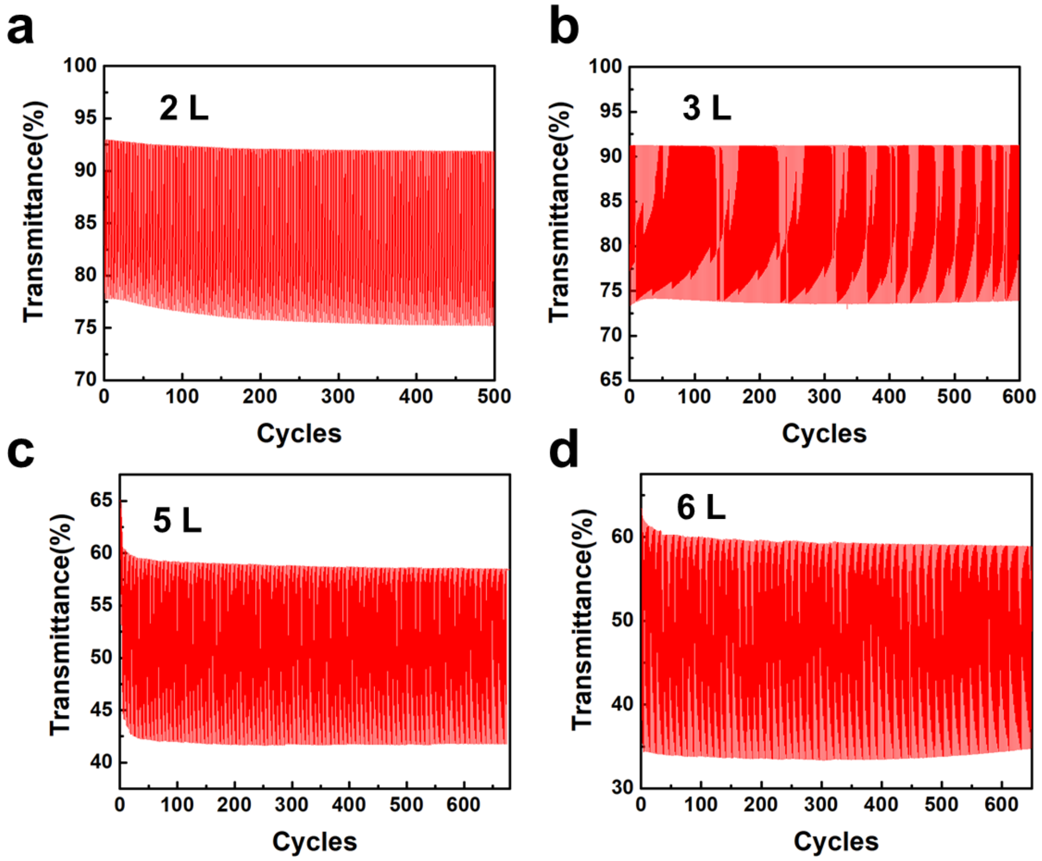

Supplementary Figure 15. Cycling stability of $TiO_2/Ti_3C_2T_x$/PET films with 2, 3, 5, and 6 layers of TiO_2 with the transmittance measured at 550 nm and the voltage changed periodically between 0 V and -1.6 V.

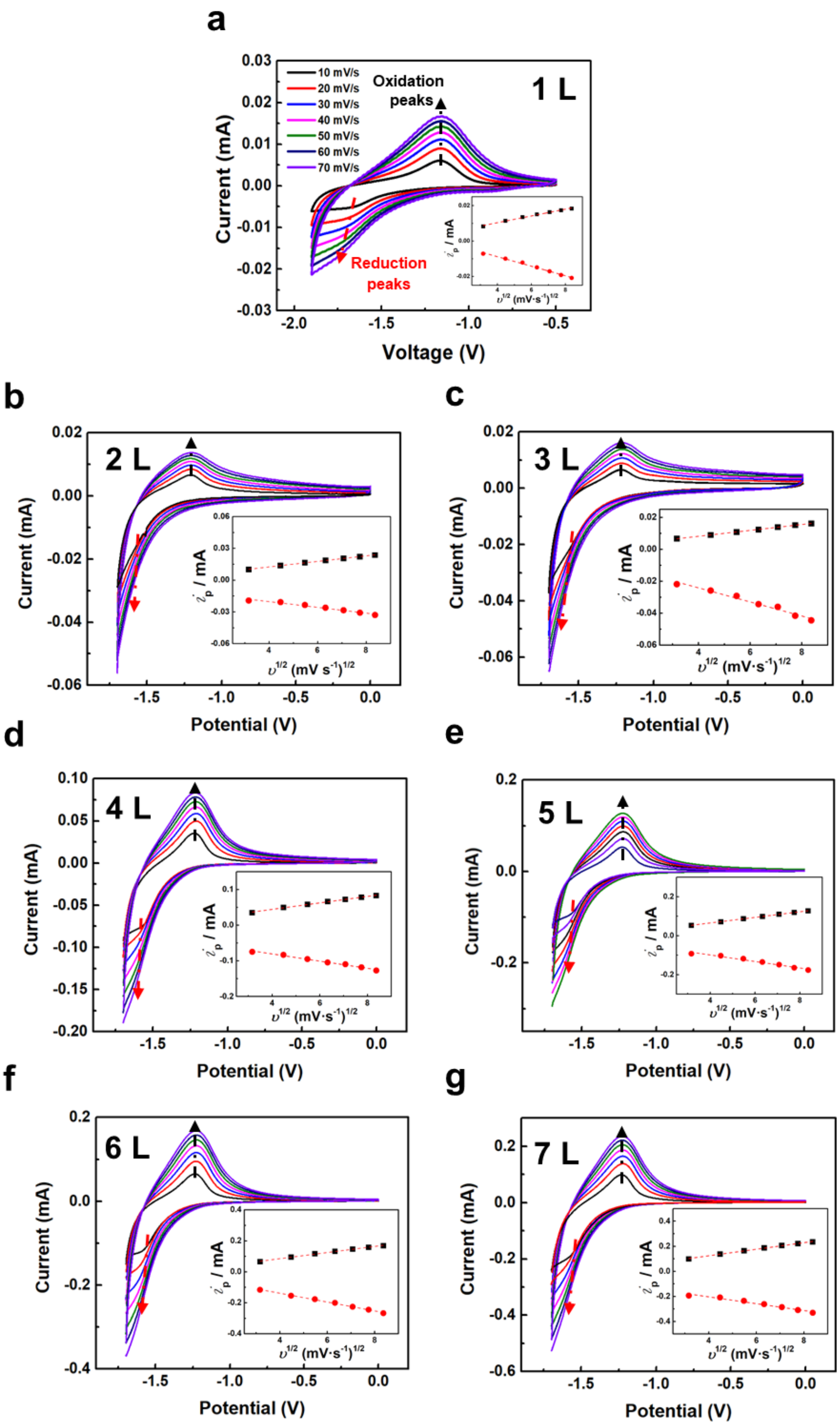

Supplementary Figure 16. Cyclic voltammetry curves of $TiO_2/Ti_3C_2T_x$/PET films. Insets: peak anodic (black) and cathodic (red) currents (i_p) as functions of square roots of scan rates ($v^{1/2}$).

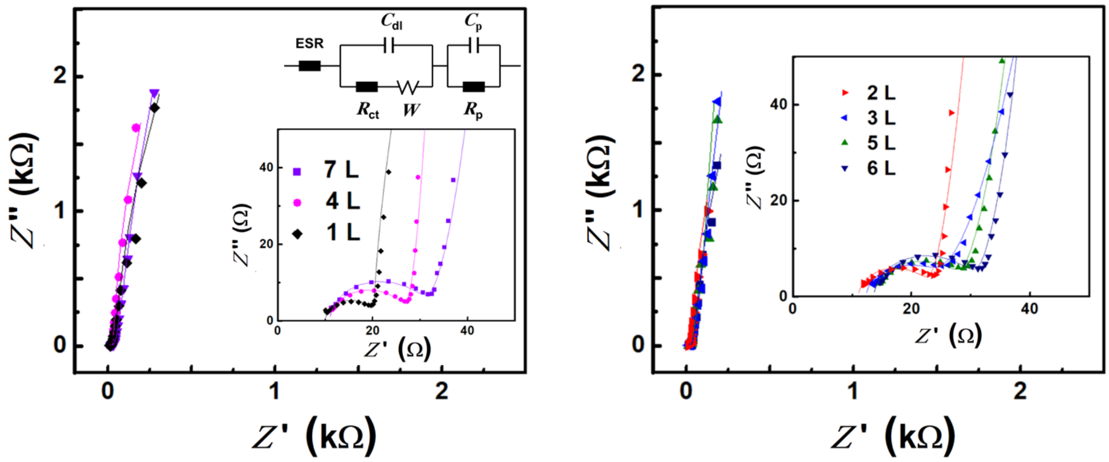

Supplementary Figure 17. Nyquist plots and corresponding simulation results (fitting lines) of $TiO_2/Ti_3C_2T_x$/PET films. Insets: equivalent circuit and zoomed-in Nyquist plots.

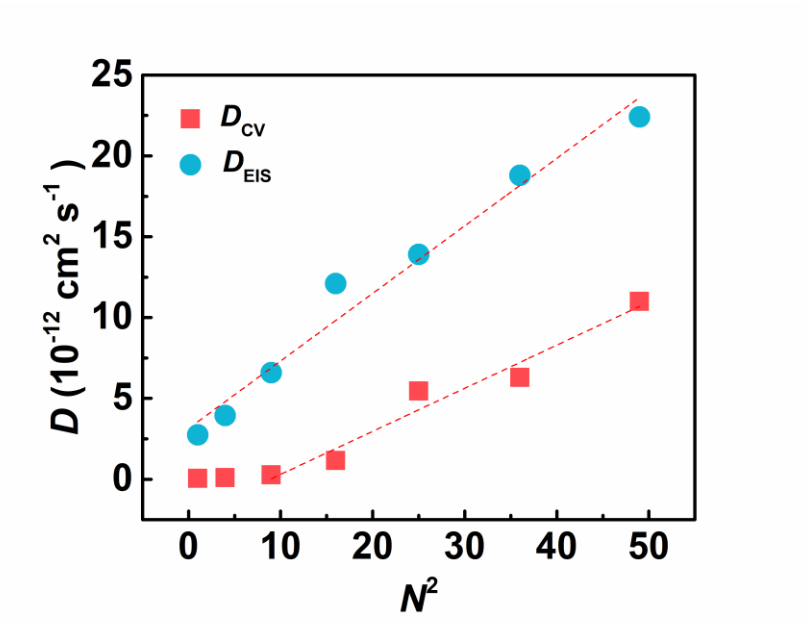

Supplementary Figure 18. Apparent ion diffusion coefficient (D) as a function of the square of the number of layers (N^2).

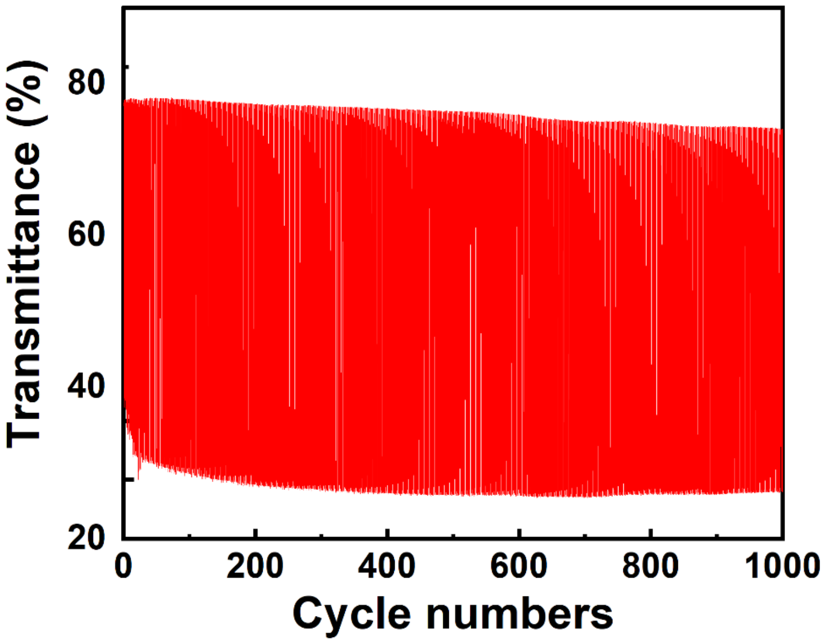

Supplementary Figure 19. Cycling stability of the flexible electrochromic device with the transmittance measured at 550 nm and the voltage changed periodically between -3 V and 1V.

Supplementary Table 1. Comparison of the sheet resistance and transmittance of transparent electrodes made with MXene, graphene, and reduced graphene oxides.

Sample/Preparing method	Sheet resistance (Ω sq^{-1})	Transmittance (%)	Reference
$Ti_3C_2T_x$/Spin coating	6	12	(*1*)
	11	28	
	25	44	
	42	54	
	51	52	
	74	62	
	106	75	
	109	69	
	200	86	
	370	91	
	1031	94	
	1975	98	
Ti_2CT_x/Spin coating	128	65	(*2*)
	507	80	
	1100	86	
	6440	96	
$Ti_3C_2T_x$-Carbon nanotube/LB	2960	88	(*3*)
	817	65	
$Ti_3C_2T_x$/Dip coating	40	51	(*4*)
	4300	94	
$Ti_3C_2T_x$/Self-assembly	3716.7	82	(*5*)
	280.83	79	
$Ti_3C_2T_x$/Spray coating	641.84	51	(*6*)
	858.33	56	
	1273.7	68	
	7974	82	
	49167	91	
$Ti_3C_2T_x$/Spin coating	470.92	77	(*7*)
	642.37	77	
	682.1	79	
	611.32	79	
	649.42	81	
	871.75	81	
	856.04	83	
Reduced graphene oxide	330	60	(*8*)
Graphene	10492	80	(*9*)
$Ti_3C_2T_x$/LLIA	1623	97	This work
	210	94	
	140	91	
	123	88	
	116	82	
	103	80	
	66	64	

Supplementary Table 2. Summary of EC properties of $TiO_2/Ti_3C_2T_x$/PET films with 1 to 7 layers of TiO_2.

**Layers**	**T_b at 550 nm (%)**	**T_c at 550 nm (%)**	**ΔT (%)**	**Coloring/bleaching time (s)**	**CE (cm^2 C^{-1})**
1	96.45	88.56	7.89	0.71/0.12	277
2	95.43	83.17	12.26	0.73/0.12	271
3	93.23	72.11	21.12	1.03/0.20	260
4	92.12	66.52	25.60	1.08/0.24	263
5	91.28	59.16	32.12	1.08/0.26	259
6	90.83	52.63	38.20	1.23/0.29	245
7	89.42	48.33	41.09	1.63/0.38	243

Supplementary Table 3. Comparison of EC properties for TiO_2 and other inorganic/organic materials.

Material	Base[a]	Structure	Thickness (nm)	T_b/T_c (%/%)	$\Delta\alpha$ (10^{-4} nm^{-1})	Coloring/ bleaching time (s)	Wavelength (nm)	CE (cm^2 C^{-1})	Cyclability (%/cycles)
TiO_2	Glass(10)	Nanowire	600	75/47	7.8	11.3/14.3	600	14	>20
	Glass(11)	Dense film	230	65/10	81.4	>300	510	56	NA
	Glass(12)	Nanoparticle	1100	2	6.3	69/7	550	18	90/2000
	Glass(13)	Nanorod	600	80/40	11.6	27/2	800	11	92/200
	Glass(14)	Nanoneedle	650	78/11	30.1	11.3/5.1	600	226	89/250
	Glass(15)	Nanorod	1000	70/42	5.1	27/5	800	16	>90/200
	PET/ **This work**	2D Flake	<50 (7 L)	89/48	123.4	1.6/0.4	550	243	>95/500
Ta-Doped TiO_2	Glass(16)	Nanoparticle	1200	87/1	37.2	66.8/6.9	550	33.2	86.3/2000
Viologen /TiO_2	PET(17)	NA	2300	75/10	8.7	8/6	600	226	87/1000
MOF	Glass(18)	Nanorod	1000	81/18	15.0	2.1/1.9	720	260	91/500
WO_3	Glass(19)	Nanosheet	650	74/13	26.8	10.7/7.0	650	NA	94/1000
	PET(20)	Nanowire	NA	70/2	NA	12.3/9	632	34.5	>1000
Polyimide	Glass(21)	Dense film	730	98/1	62.8	20/20	796	99	40/5000
Viologen	PET(22)	Dense film	NA	98/70	NA	26/39	550	65	53/75
PEDOT	PET(23)	NA	NA	24	NA	1.5/0.9	550	375	95/4000
	PET(24)	Dense film	407	63/4	29	8/10	620	530	91/10000
	PET (25)	Dense film	NA	38/9	NA	0.3/2.5	550	391	NA
PANI	Glass(26)	Dense film	60	59/75	17	1.4/1.4	700	46	90/300
	Glass(27)	Nanowire	NA	19/82	NA	1.0/ 1.5	550	206	500
Poly-Pyrrole	Glass(28)	Dense film	NA	84/23	NA	~2	565	697	NA

a: reference.

Supplementary Table 4. Fitted parameters according to electrochemical impedance spectroscopy of $TiO_2/Ti_3C_2T_x$/PET films with 1 to 7 layers of TiO_2.

Layers	*ESR* (Ω)	R_{ct} (Ω)	CPE_{dl} (μF)	CPE_p (μF)	R_p (kΩ)
1	10.1	8.7	1.8	17.6	19.6
2	10.9	8.9	2.2	19.1	19.2
3	14.1	10.8	2.5	17.4	16.9
4	13.9	12.5	2.2	17.6	21.3
5	9.8	14.9	2.8	17.4	16.8
6	9.8	15.0	2.9	18.2	16.5
7	11.6	15.7	3.1	18.6	16.9

Supplementary References

1. C. Zhang, B. Anasori, A. Seral-Ascaso, S. H. Park, N. McEvoy, A. Shmeliov, G. S. Duesberg, J. N. Coleman, Y. Gogotsi, V. Nicolosi, Transparent, Flexible, and Conductive 2D Titanium Carbide (MXene) Films with High Volumetric Capacitance. *Adv. Mater*. **29**, 1702678 (2017).
2. G. Ying, A. D. Dillon, A. T. Fafarman, M. W. Barsoum, Transparent, Conductive Solution Processed Spincast 2D Ti_2CTx (MXene) Films. *Mater. Res. Lett*. **5**, 391–398 (2017).
3. G.M. Weng, J. Li, M. Alhabeb, C. Karpovich, H. Wang, J. Lipton, K. Maleski, J. Kong, E. Shaulsky, M. Elimelech, Y. Gogotsi, A. D. Taylor, Layer-by-Layer Assembly of Cross-Functional Semi-transparent MXene-Carbon Nanotubes Composite Films for Next-Generation Electromagnetic Interference Shielding. *Adv. Funct. Mater*. **28**, 1803360 (2018).
4. P. Salles, E. Quain, N. Kurra, A. Sarycheva, Y. Gogotsi, Automated Scalpel Patterning of Solution Processed Thin Films for Fabrication of Transparent MXene Microsupercapacitors. *Small* **14**, 1802864 (2018).
5. S. J. Kim, J. Choi, K. Maleski, K. Hantanasirisakul, H.T. Jung, Y. Gogotsi, C. W. Ahn, Interfacial Assembly of Ultrathin, Functional MXene Films. *ACS Appl. Mater. Interfaces* **11**, 32320–32327 (2019).
6. K. Hantanasirisakul, M. Q. Zhao, P. Urbankowski, J. Halim, B. Anasori, S. Kota, C. E. Ren, M. W. Barsoum, Y. Gogotsi, Fabrication of $Ti_3C_2T_x$ MXene Transparent Thin Films with Tunable Optoelectronic Properties. *Adv. Electron. Mater*. **2**, 1600050 (2016).
7. M. Mariano, O. Mashtalir, F. Q. Antonio, W. H. Ryu, B. Deng, F. Xia, Y. Gogotsi, A. D. Taylor, Solution-processed Titanium Carbide MXene Films Examined as Highly Transparent Conductors. *Nanoscale* **8**, 16371–16378 (2016).
8. H. A. Becerril, J. Mao, Z. Liu, R. M. Stoltenberg, Z. Bao, Y. Chen, Evaluation of Solution-Processed Reduced Graphene Oxide Films as Transparent Conductors. *ACS Nano* **2**, 463–470 (2008).
9. J. Wu, H. A. Becerril, Z. Bao, Z. Liu, Y. Chen, P. Peumans, Organic Solar Cells With Solution-Processed Graphene Transparent Electrodes. *Appl. Phys. Lett.* **92**, 263302 (2008).

10. J. Z. Chen, W.-Y. Ko, Y. C. Yen, P. H. Chen, K. J. Lin, Hydrothermally Processed TiO_2 Nanowire Electrodes with Antireflective and Electrochromic Properties. *ACS Nano* **6**, 6633–6639 (2012).

11. N. N. Dinh, N. Th. T. Oanh, P. D. Long, M. C. Bernard, A. Hugot-Le Goff, Electrochromic Properties of TiO_2 Anatase Thin Films Prepared by A Dipping Sol–Gel Method. *Thin Solid Films* **423**, 70–76 (2003).

12. Z. Tebby, O. Babot, T. Toupance, D. H. Park, G. Campet, M. H. Delville, Low-Temperature UV-Processing of Nanocrystalline Nanoporous Thin TiO_2 Films: An Original Route toward Plastic Electrochromic Systems. *Chem. Mater.* **20**, 7260–7267 (2008).

13. Y. Chen, X. Li, Z. Bi, X. He, G. Li, X. Xu, X. Gao, Design and Construction of Hierarchical TiO_2 Nanorod Arrays by Combining Layer-by-layer and Hydrothermal Crystallization Techniques for Electrochromic Application. *Appl. Surf. Sci*. **440**, 217–223 (2018).

14. R. A. Patil, R. S. Devan, Y. Liou, Y. R. Ma, Efficient Electrochromic Smart Windows of One-Dimensional Pure Brookite TiO_2 Nanoneedles. *Sol. Energy Mater. Sol. Cells* **147**, 240–245 (2016).

15. Y. Chen, X. Li, Z. Bi, X. He, X. Xu, X. Gao, Core-Shell Nanorod Arrays of Crystalline/Amorphous TiO_2 Constructed by Layer-by-Layer Method for High-Performance Electrochromic Electrodes. *Electrochim. Acta*. **251**, 546–553 (2017).

16. S. Cao, S. Zhang, T. Zhang, J. Y. Lee, Fluoride-Assisted Synthesis of Plasmonic Colloidal Ta-Doped TiO_2 Nanocrystals for Near-Infrared and Visible-Light Selective Electrochromic Modulation. *Chem. Mater.* **30**, 4838–4846 (2018)

17. Y. Alesanco, J. Palenzuela, R. Tena-Zaera, G. Cabañero, H. Grande, B. Herbig, A. Schmitt, M. Schott, U. Posset, A. Guerfi, M. Dontigny, K. Zaghib, A. Viñuales, Plastic Electrochromic Devices Based On Viologen-modified TiO_2 Films Prepared at Low Temperature. *Sol. Energy Mater. Sol. Cells* **157**, 624–635 (2016).

18. R. Li, K. Li, G. Wang, L. Li, Q. Zhang, J. Yan, Y. Chen, Q. Zhang, C. Hou, Y. Li, H. Wang, Ion-Transport Design for High-Performance Na^+-Based Electrochromics. *ACS Nano* **12**, 3759–3768 (2018).

19. A. Azam, J. Kim, J. Park, T. G. Novak, A. P. Tiwari, S. H. Song, B. Kim, S. Jeon, Two-Dimensional WO_3 Nanosheets Chemically Converted from Layered WS_2 for High-Performance Electrochromic Devices. *Nano Lett.* **18**, 5646–5651 (2018).

20. J. L. Wang, Y. R. Lu, H. H. Li, J. W. Liu, S. H. Yu, Large Area Co-Assembly of Nanowires for Flexible Transparent Smart Windows. *J. Am. Chem. Soc.* **139**, 9921–9926 (2017).

21. Q. Zhang, C. Y. Tsai, L. J. Li, D. J. Liaw, Colorless-to-Colorful Switching Electrochromic Polyimides with Very High Contrast Ratio. *Nat. Commun.* **10**, 1239 (2019).

22. E. Hwang, S. Seo, S. Bak, H. Lee, M. Min, H. Lee, An Electrolyte-Free Flexible Electrochromic Device Using Electrostatically Strong Graphene Quantum Dot-Viologen Nanocomposites. *Adv. Mater*. **26**, 5129–5136 (2014).

23. R. Singh, J. Tharion, S. Murugan, A. Kumar, ITO-Free Solution-Processed Flexible Electrochromic Devices Based on PEDOT:PSS as Transparent Conducting Electrode. *ACS Appl. Mater. Inter.* **9**, 19427–19435 (2016).

24. J. Jensen, M. Hösel, , I. Kim, J. Yu, J. Jo, C. Krebs, Fast Switching ITO Free Electrochromic Devices.

Adv. Funct. Mater. ***24***, 1228–1233 (2014).

25. S. Macher, M. Schott, M. Sassi, I. Facchinetti, R. Ruffo, G. Patriarca, L. Beverina, U. Posset, A. Giffin, P. Löbmann, New Roll-to-Roll Processable PEDOT-Based Polymer with Colorless Bleached State for Flexible Electrochromic Devices. *Adv. Funct. Mater.* **30**, 1906254 (2019).
26. L. Zhao, L. Zhao, Y. Xu, T. Qiu, L. Zhi, G. Shi, Polyaniline Electrochromic Devices With Transparent Graphene Electrodes. *Electrochim Acta.* **55**, 491–497 (2009).
27. S. Zhang, G. Sun, Y. He, R. Fu, Y. Gu, S. Chen, Preparation, Characterization, and Electrochromic Properties of Nanocellulose-based Polyaniline Nanocomposite Films. *ACS Appl. Mater. Inter.* **9**, 16426–16434 (2017).
28. T. Soganci, S. Soyleyici, H. C. Soyleyici, M. Ak. High Contrast Electrochromic Polymer and Copolymer Materials Based on Amide-substituted Poly(dithienyl pyrrole). *J. Electrochem. Soc.* **164**, H11 (2016).
