# Supplementary material for: Flexible and high-performance electrochromic devices enabled by self-assembled 2D TiO2/MXene heterostructures
Source: Nat Commun. 2021 Mar 11;12:1587. doi: 10.1038/s41467-021-21852-7 (PMC7952574; doi:10.1038/s41467-021-21852-7)
Supplement: Supplementary file 2 — Description of Additional Supplementary Files [file 41467_2021_21852_MOESM2_ESM.docx]

**Supplementary Data:** Supplementary Figure 1 to Figure 19, Supplementary Table 1 to Table 4

**Supplementary Movie 1:** Real-time movie of the Ti3C2Tx flakes self-assembly at liquid/liquid interface on a laboratory scale.

**Supplementary Movie 2:** Real-time movie of the TiO2 flakes self-assembly at liquid/liquid interface on a laboratory scale.
